# Dynamic changes in the plastid and mitochondrial genomes of the angiosperm *Corydalis pauciovulata* (Papaveraceae)

**DOI:** 10.1186/s12870-024-05025-4

**Published:** 2024-04-22

**Authors:** Seongjun Park, Boram An, SeonJoo Park

**Affiliations:** 1 Institute of Natural Science, Yeungnam University, Gyeongsan, Gyeongbuk 38541 South Korea; 2 Department of Life Sciences, Yeungnam University, Gyeongsan, Gyeongbuk 38541 South Korea

**Keywords:** DNA-RRR, Genome rearrangement, Organelle genomes, Concomitant loss, NDH complex

## Abstract

**Background:**

*Corydalis* DC., the largest genus in the family Papaveraceae, comprises > 465 species. Complete plastid genomes (plastomes) of *Corydalis* show evolutionary changes, including syntenic arrangements, gene losses and duplications, and IR boundary shifts. However, little is known about the evolution of the mitochondrial genome (mitogenome) in *Corydalis*. Both the organelle genomes and transcriptomes are needed to better understand the relationships between the patterns of evolution in mitochondrial and plastid genomes.

**Results:**

We obtained complete plastid and mitochondrial genomes from *Corydalis pauciovulata* using a hybrid assembly of Illumina and Oxford Nanopore Technologies reads to assess the evolutionary parallels between the organelle genomes. The mitogenome and plastome of *C. pauciovulata* had sizes of 675,483 bp and 185,814 bp, respectively. Three ancestral gene clusters were missing from the mitogenome, and expanded IR (46,060 bp) and miniaturized SSC (202 bp) regions were identified in the plastome. The mitogenome and plastome of *C. pauciovulata* contained 41 and 67 protein-coding genes, respectively; the loss of genes was a plastid-specific event. We also generated a draft genome and transcriptome for *C. pauciovulata*. A combination of genomic and transcriptomic data supported the functional replacement of acetyl-CoA carboxylase subunit β (*accD*) by intracellular transfer to the nucleus in *C. pauciovulata*. In contrast, our analyses suggested a concurrent loss of the NADH-plastoquinone oxidoreductase (*ndh*) complex in both the nuclear and plastid genomes. Finally, we performed genomic and transcriptomic analyses to characterize DNA replication, recombination, and repair (DNA-RRR) genes in *C. pauciovulata* as well as the transcriptomes of *Liriodendron tulipifera* and *Nelumbo nuicifera*. We obtained 25 DNA-RRR genes and identified their structure in *C. pauciovulata*. Pairwise comparisons of nonsynonymous (*d*_N_) and synonymous (*d*_S_) substitution rates revealed that several DNA-RRR genes in *C. pauciovulata* have higher *d*_N_ and *d*_S_ values than those in *N*. *nuicifera.*

**Conclusions:**

The *C. pauciovulata* genomic data generated here provide a valuable resource for understanding the evolution of *Corydalis* organelle genomes. The first mitogenome of Papaveraceae provides an example that can be explored by other researchers sequencing the mitogenomes of related plants. Our results also provide fundamental information about DNA-RRR genes in *Corydalis* and their related rate variation, which elucidates the relationships between DNA-RRR genes and organelle genome stability.

**Supplementary Information:**

The online version contains supplementary material available at 10.1186/s12870-024-05025-4.

## Background

Mitochondria and plastids originate from alphaproteobacterial and cyanobacterial endosymbionts, respectively [1, 2]. The genomes of both are highly reduced relative to the ancestral genome because substantial numbers of genes were lost, and many essential genes were transferred into the nuclear genome of a host cell over evolutionary time [3]. In angiosperms, the mitochondrial and plastid genomes (mitogenomes and plastomes) are critical in respiration and photosynthesis, encoding only 41 and 79 proteins, respectively [4, 5]. Thus, coordination between nuclear-encoded organelle-targeted and organelle-encoded proteins is essential for their function [6]. This process involves the import of nuclear-encoded organelle-targeted proteins, which contributes to organelle genome stability [7]; DNA replication, recombination, and repair (RRR) system [8]; posttranscriptional regulation, and translation initiation [9]. Many nuclear-encoded organelle-targeted proteins are dual-targeted to mitochondria and plastids [10]. Dysfunction of DNA-RRR genes, such as *RECA* and *MSH1*, has been suggested to be a mechanism for rate acceleration of angiosperm organelle genomes [11, 12]. These genes also regulate recombination activity in mitogenomes [13, 14]. Researchers examined the relationship between dysfunction in DNA-RRR systems and plastome complexity in Geraniaceae and revealed a significant correlation between substitution rates and three DNA-RRR genes (*GYRA*, *WHY1*, and *UVRB/C*) [15]. Thus, a comprehensive understanding of organelle genome evolution in plants requires a combination of organelle genomics and transcriptomics approaches.

The mitogenome and plastome of angiosperms vary in size, structure, and gene content, although the organelle genomes exhibit parallel evolutionary relics. For example, angiosperm mitogenomes range from 65.7 kb in *Viscum scurruloideum* [16] to 11.3 Mb in *Silene conica* [12], containing variable protein-coding genes ranging from 19 in *V. scurruloideum* [16] to 41 in *Liriodendron tulipifera* [17]. They exhibit multipartite organization, mapping as circular, linear, or branched molecules due to active recombination associated with repeats [18]. In contrast, angiosperm plastomes generally exhibit a circular quadripartite structure with large single-copy (LSC) and small single-copy (SSC) regions separated by two copies of an inverted repeat (IR) region, varying from 11.3 kb to 242.5 kb in size with 5–79 protein-coding genes [5, 19]. However, the plastomes of some lineages of angiosperms exhibit structural changes, including IR loss and genome rearrangements [20]. In both genomes, several organelle genes have been successfully transferred to the nucleus through direct intracellular gene transfer (IGT) or substitution by a nuclear homolog [21]. In addition to IGT to the nucleus, intercompartmental transfers between organellar counterparts have been observed (mitochondrial DNA of plastid origin, MIPTs; plastid DNA of mitochondrial origin, PLMTs) [22]. MIPTs are a common feature of the mitogenome in angiosperms, while PLMTs are rare.

The genus *Corydalis* DC. consists of annual or perennial herbaceous plants and belongs to Papaveraceae Juss. It comprises approximately 465 species distributed throughout the Northern Hemisphere and tropical eastern Africa [23]. The plastomes of 72 *Corydalis* species have been sequenced (the NCBI database, accessed on May 24, 2023), representing only 15.5%. The sequenced *Corydalis* plastomes ranged in size from 149.9 kb in *C. mucronifera* (BK063233) to 218.8 kb in *C. hendersonii* (OP747311) with a quadripartite organization. The variation in plastome sizes within the genus is due to IR expansions, ranging from 22.7 kb to 54.9 kb. The *Corydalis* plastomes also exhibit divergent structural evolution, including multiple inversions and gene losses [24–27]. In particular, the losses of acetyl-CoA carboxylase subunit β (*accD*), ATP-dependent Clp protease proteolytic subunit gene (*clpP*), or all 11 subunits of NADH-plastoquinone oxidoreductase (*ndh*) are lineage-specific events within the genus [27, 28].

*Corydalis* organelle genomes can provide excellent examples for studying the evolution of genome architecture, gene losses, mutation rates, and cytonuclear interactions. However, no complete mitogenome has been assembled and analyzed for the genus *Corydalis*, even at the level of the family Papaveraceae. We also have limited knowledge about DNA-RRR proteins in the *Corydalis* nuclear genome. In this study, we sequenced, assembled, and analyzed the complete sequences of the plastid and mitochondrial genomes of *C. pauciovulata* Ohwi and generated a draft nuclear genome and transcriptome. *Corydalis pauciovulata* Ohwi is an annual or biennial herb native to moist regions near streams and mountain valleys in Korea and Japan [29]. Our purpose of this study was to 1) explore the evolutionary characteristics of the *C. pauciovulata* plastid and mitochondrial genomes, 2) determine the nuclear-encoded DNA-RRR proteins, 3) identify the evolutionary fate of the lost genes in the organelle genomes, and 4) understand the driving factors of the dynamic genomic features of *C. pauciovulata* organelle genomes. For do that, we compared them to those of *Nelumbo nucifera* (since none of the *Corydalis* has a published mitogenome), as well as *L. tulipifera* as an outgroup, for which both organelle genomes and the transcriptome are available, to better understand the evolution of gene content, structure, and substitution rates.

## Results

### Organelle genome assemblies and genome organization

**Fig. 1:**
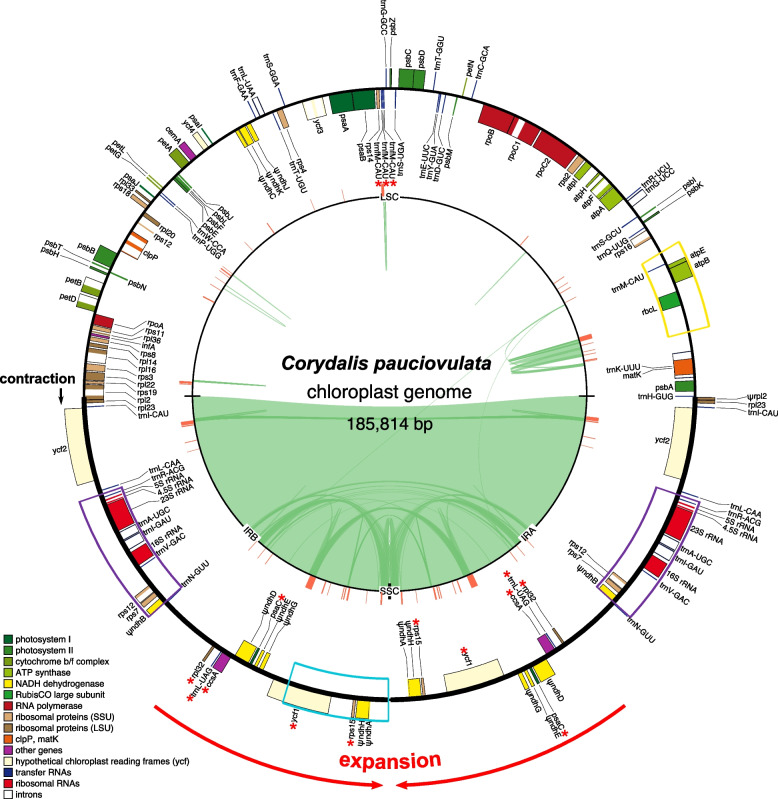
The *Corydalis pauciovulata* plastome. Thick lines on the genome map indicate the inverted repeats (IRa and IRb: 46,060 bp), which separate the genome into small (SSC: 202 bp) and large (LSC: 92,155) single-copy regions. Genes on the inside and outside of the map are transcribed in clockwise and counterclockwise directions, respectively. Asterisks indicate genes transferred from single-copy regions to the IR, and ψ denotes a pseudogene. The red lines on the inner circle indicate tandem repeats. The black and red arrows on the outside of the map indicate contraction and expansion events, respectively. The colored boxes on the map correspond to the locally collinear blocks inferred by Mauve (see Fig. 3). The green lines within the inner circle indicate the positions of the pairs of repeats, with crossed connecting lines denoting reverse repeats

**Fig. 2:**
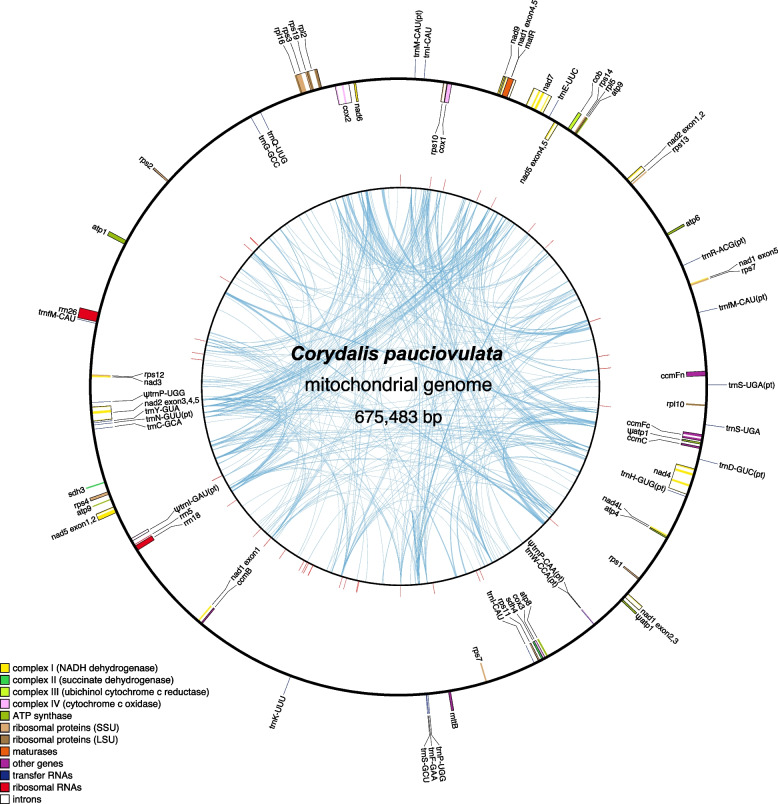
The *Corydalis pauciovulata* mitogenome. Genes on the inside and outside of the map are transcribed in clockwise and counterclockwise directions, respectively. The red lines on the inner circle indicate tandem repeats, and ψ denotes a pseudogene. The blue lines within the inner circle indicate the positions of the pairs of repeats, with crossed connecting lines denoting reverse repeats

The newly sequenced plastid and mitochondrial genomes of *C. pauciovulata* were assembled into circular molecules with lengths of 185,814 bp and 675,483 bp, respectively (Table 1 and Figs. 1 and 2). Depth of coverage analyses revealed that the organelle genomes were deeply (PE/MP/ONT; plastome: 3,092 × /1,932 × /280 × , mitogenome: 170 × /155 × /26 ×) covered (Figure S1), supporting the accuracy of the assemblies.

**Table 1:** General features of *Corydalis pauciovulata* organelle genomes

	Plastome	Mitogenome
Genome size (bp)	185,814	675,483
LSC (bp)	93,492	-
IR (bp)	46,060	-
SSC (bp)	202	-
GC content (%)	41.3	46.1
Protein genes	67	41
rRNA genes	4	3
tRNA genes	29	12
*plastid-derived*	-	10
Introns		
*cis*	17	21
*trans*	1	5
Repeat content (%)	7.53	5.55

**Fig. 3:**
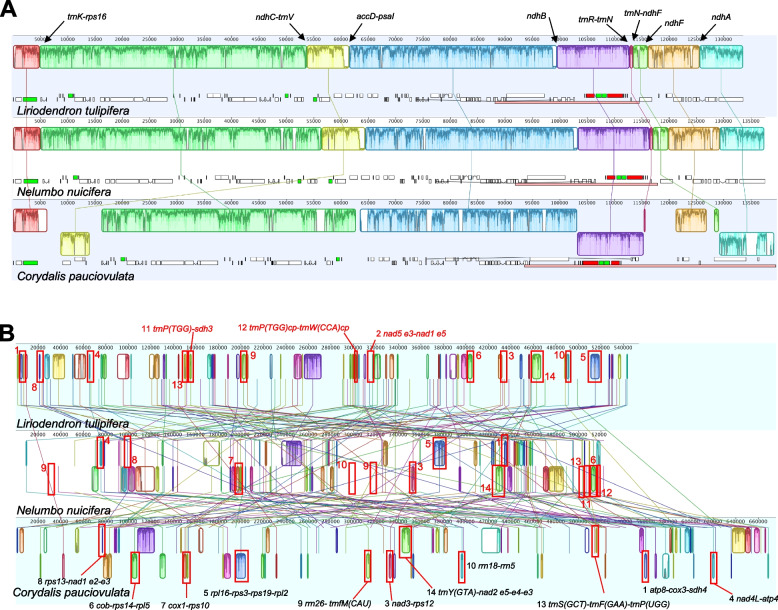
Structural alignments of the organelle genome arrangements in *Corydalis pauciovulata*. Blocks drawn below the horizontal line indicate sequences found in an inverted orientation. **A** The colored blocks represent collinear sequence blocks shared by all plastomes. Individual genes and strandedness are represented below the *Liriodendron* genome block. Only one copy of the inverted repeat (IR) is shown for each plastome, and the pink box below each plastome block indicates its IR. **B** The colored blocks represent collinear sequence blocks shared by all mitogenomes. The red boxes indicate the conserved gene clusters

The *C. pauciovulata* plastome had a general quadripartite structure; however, it contained expanded IR (46,060 bp) and miniaturized SSC (202 bp) regions (Fig. 1). An analysis of genome rearrangements with *L. tulipifera* and *N. nucifera* suggested that the *C*. *pauciovulata* plastome has experienced three inversions with eight breakpoints: *trnK-rps16*, *ndhC-trnV*, *accD-psaI*, *ndhB*, *trnR-trnN*, *trnN-ndhF*, *ndhF,* and *ndhA* (Fig. 3A). The first inversion (yellow box) with the *rbcL-atpB-atpE-trnM* region was relocated (Figs. 1 and 3A). Compared to the published *L. spectabilis* plastome, which is from a related genus in the same subfamily, the second inversion (purple box) involving a pair of breakpoints (*ndhB* and *trnR-trnN*) in the IR region suggests a lineage-specific event (Fig. 1 and Figure S2). The third inversion (blue box) with the *ycf1-rps15-ndhH-ndhA* region was the result of the expansion of the IR_B_ (Figs. 1 and 3A).

**Fig. 4:**
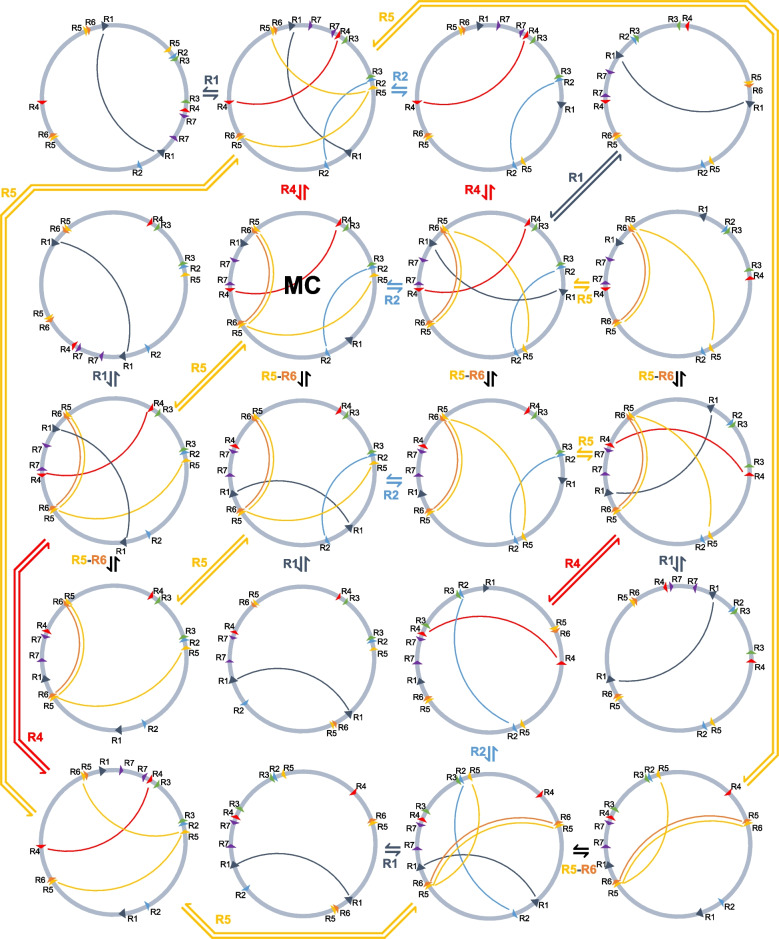
Mitogenome rearrangements in *Corydalis pauciovulata*. Alternative genomic conformations based on five repeat pairs (R1, R2, R4, R5, and R6). MC: master circle corresponding to the mitogenome in Fig. 2

The *C. pauciovulata* mitogenome showed high levels of structural divergence in comparison to the *L. tulipifera* and *N. nucifera* mitogenomes (Fig. 3B). However, 11 conserved gene clusters were present in the *C. pauciovulata* mitogenome among the 14 ancestral gene clusters. Three ancestral gene clusters were missing in *Corydalis*: *nad5* exon 3-*nad1* exon 5, *sdh3-trnP-UGG*, and *trnP-UGG(cp)-trnW-CCA(cp)* (Fig. 3B). In the *N. nucifera* conserved gene clusters, only one gene cluster was missing (*nad5* exon 3-*nad1* exon 5; Fig. 3B). The *C. pauciovulata* mitogenome contained 459 repeat pairs, including three large (> 1 kb), 77 intermediate (100–1000 bp), and 379 small (< 100 bp) repeats (Table 1 and Fig. 2). Among these repeats, seven repeat pairs (R1 to R7) were identified as potentially recombinationally active based on a thorough analysis of corrected long reads and other contigs (Figure S3). These contigs displayed conflicts with the master circle and spanned predicted recombination boundaries, providing evidence to support the determination of their recombination activity. Assuming recombination across each IR (excluding R3 and R7), 19 additional genomic conformations could be predicted (Fig. 4), all containing the same genomic information.

### Gene content in organelle genomes

The *C. pauciovulata* plastome contains 67 proteins, 29 tRNAs, and four rRNAs (Table 1 and Table S1). The functions of all 11 NADH-plastoquinone oxidoreductases (*ndh*) in the plastome were lost due to frameshift mutations (*ndhJ*, *ndhK*, and *ndhG*), premature stop codons (*ndhC*, *ndhD*, *ndhE*, and *ndhH*), degradation (*ndhA* and *ndhB*), or complete loss (*ndhI* and *ndhF*). In addition to the functional loss of the 11 *ndh* genes, the *accD* and *trnV-UAC* genes were also absent from the *C. pauciovulata* plastome. Multiple genes were duplicated, including sequences from the *rpl32*, *trnL-UGA*, *ccsA*, *ψndhD*, *psaC*, *ψndhE*, *ψndhG*, *ycf1*, *rps15*, *ψndhH*, and *ψndhA* genes, due to IR boundary shifts (Fig. 1). Triplication of *trnfM-CAU* was observed in the *C. pauciovulata* plastome (Fig. 1). The plastome of *C. pauciovulata* contained 99 repeat pairs, covering 7.53% of the genome (Table 1 and Fig. 1).

The *C. pauciovulata* mitogenome contained a full set of 41 protein-coding genes, 12 tRNAs, and three rRNAs (Table 1). Twelve plastid-derived tRNAs were identified, and two of those tRNAs were pseudogenes (Table 1 and Fig. 2). Two copies of *rps7, trnP-UGG,* and *trnI-CAU* were identified in the *C. pauciovulata* mitogenome, but one copy of *trnP-UGG* appeared to be a pseudogene (Fig. 2). Thirty-six MIPTs were identified in the *C. pauciovulata* mitogenome, ranging from 64 to 6,500 bp and covering 4.21% of the genome (Table S2). PREP-Mt predicted 738 putative C-to-U RNA editing sites to 41 *C. pauciovulata* mitochondrial protein-coding genes, more than in *N. nucifera* (715 sites) but fewer than in *L. tulipifera* (784 sites) (Table S3). The available transcriptome data for 21 mitochondrial genes revealed 357 sites, and of the 328 sites predicted by PREP-Mt for these genes, 299 (91%) were edited (Table S4). Nine hundred thirteen ORFs (≥ 150 bp in length) were identified in intergenic regions of the *C. pauciovulata* mitogenome. CD-search identified several ORFs harboring a partial or intact sequence homologous to RNase H (*Ty1/Copia* and *Ty3/Gypsy*), integrase, reverse transcriptase, mitovirus RNA-dependent RNA polymerase, DNA polymerase type B, and endonuclease/exonuclease/phosphatase families (Table S5). Twelve ORFs (≥ 150 bp in length) were identified that contained small fragments (> 30 bp) of one or two mitochondrial genes (e.g., *atp1*, *rps19*, *rpl2*, *rpl5*, *ccmFc*, *rps7*, *cob*, *nad5*, *sdh3*, and *sdh4*) (Table S6). Seven of these ORFs (*orf457a/b*, *orf244*, *orf234, orf146, orf56*, and *orf54*) were predicted to encode one or three transmembrane helices (Table S6). Among them, *orf457a/b* was immediately downstream from a repeat (R1) that overlapped with the *atp1* gene; the first 701 bp of *orfs* and *atp1* were identical. Multiple transcripts had a sequence identical to that of the ORFs (Table S6 and Figure S4). The *orf146* that contained a fragment of *rpl5* was also associated with repeats (R5-R6) and was upstream of *rps2* and *orf244* (Figure S4).

### Evolutionary fate of organelle genes

**Fig. 5:**
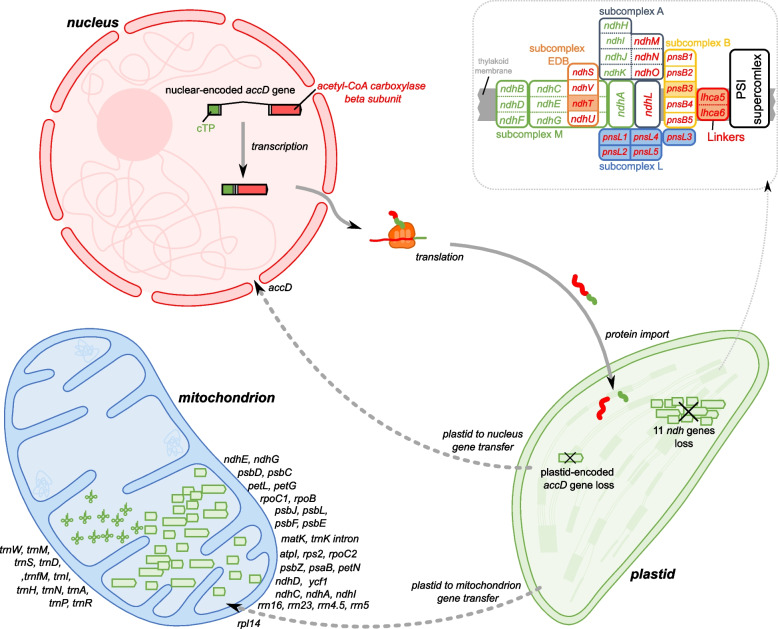
Schematic diagram of the organelle gene transfer to the nucleus and the NDH-PSI supercomplex. The colored blocks represent collinear sequence blocks shared by all plastomes. Blocks drawn below the horizontal line indicate sequences found in an inverted orientation. Individual genes and strandedness are represented below the *Euptelea* genome block. Only one copy of the inverted repeat (IR) is shown for each plastome, and the pink box below each plastome block indicates its IR

To identify potential organelle-to-nucleus functional transfers (including an intermediate stage), we assembled a de novo transcriptome of *C*. *pauciovulata*. The completeness of the gene sets was assessed using BUSCO with the eudicot database of 2,236 conserved genes: 89.9% had complete gene coverage, 2.3% were fragmented, and only 7.8% were missing (Figure S5). All 79 plastid and 41 mitochondrial protein-coding genes were used to query the *C*. *pauciovulata* transcriptome. We found a nuclear-encoded *accD*-like ORF with 88.8% nucleotide sequence identity to the *Lamprocapnos spectabilis* plastid *accD* gene. TargetP predicted the first 84 amino acids of this ORF to be a cTP (chloroplast = 0.975). PCR and Sanger sequencing identified the nuclear-encoded plastid-targeted *ACCD*, and the nucleotide sequence alignments of both copies confirmed the presence of an intron (Fig. 5 and Figure S6). Using all 11 plastid *ndh* gene sequences from the *L. spectabilis* plastome as BLAST queries, we found no *ndh*-like gene sequences in the *C*. *pauciovulata* transcriptome. To address potential parallel loss of the plastid-encoded *ndh* genes and nuclear-encoded NDH-related genes in *C. pauciovulata*, we queried the amino acid sequences of the nuclear-encoded NDH-related protein complexes (Table S7) with the translated *C*. *pauciovulata* transcriptome. Among the 20 nuclear NDH-related genes, we found only the nuclear-encoded *ndhT* from subcomplex EDB, *pnsB3* from subcomplex B, all subcomplex L genes (*psnL1*-*5*), and two linkers (*lhca5* and *lhca6*) (Fig. 5).

Substitution of the duplicated nuclear *ACCase*, *RPL20*, *RPL23*, and *RPS16* gene sequences for the plastids was not detected in the *C*. *pauciovulata* transcriptome; one copy of a cytosolic homolog of eukaryotic (*ACCase*) and mitochondrial (*RPS16*) origin was identified; and two copies of a cytosolic homolog of eukaryotic (*RPL23*) and mitochondrial (*RPL20*) origin were identified, but no transit peptides were predicted (Figure S7).

### Identification and characterization of nuclear DNA-RRR genes

**Fig. 6:**
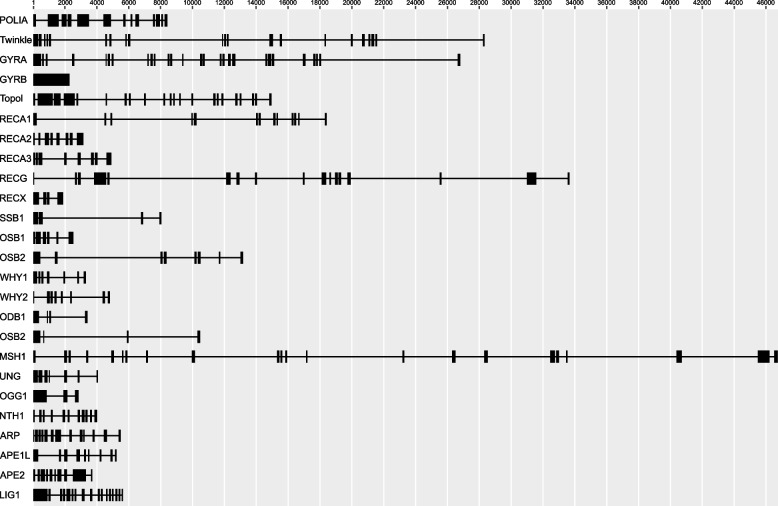
Structure of 25 DNA replication, recombination, and repair system genes in *Corydalis pauciovulata*. Exons and introns are represented by boxes and lines, respectively

To identify *C*. *pauciovulata* DNA-RRR genes, we queried the amino acid sequences of the 32 selected DNA-RRR genes from *A. thaliana*, which were classified into nine categories (Table S8), with the translated *C*. *pauciovulata* transcriptome. A total of 25 DNA-RRR transcripts were identified in the transcriptome data (Table S8). We failed to find seven DNA-RRR genes, *POLIB*, *GYRBM*, *SSB2*, *OSB3*, *OSB 4*, *WHY3*, or *NTH2*. The predicted ORF sizes of the DNA-RRR genes ranged from 612 bp in *ODB1* to 3,618 bp in *Topol*. We assembled a draft nuclear genome to determine the structure of DNA-RRR genes from *C*. *pauciovulata*. The frequency of 21-mers in the Illumina data was calculated using Jellyfish followed by GenomeScope (Figure S8). The proportion of homozygosity in *C*. *pauciovulata* was evaluated to be 99.2%, and the genome size was estimated to be 236.3 Mb (Figure S8). The hybrid genome assembly (PE, MP, and ONT reads) generated a draft nuclear genome of *C*. *pauciovulata* containing 3,821 contigs with a total length of 203.3 Mb. The completeness of the draft nuclear genome was also assessed using BUSCO with the eudicot database: 90.9% had complete gene coverage, 3.2% were fragmented, and only 5.9% were missing (Figure S8). The 25 DNA-RRR CDSs of *C*. *pauciovulata* were used as queries in “BLASTN” against the draft de novo nuclear genome sequence of *C*. *pauciovulata*. Available nuclear genome data for 25 genes confirmed the exon/intron patterns of the *Corydalis* DNA-RRR genes (Fig. 6). The number of exons in 25 genes ranged from one (*GYRBC*) to 27 (*GYRA*).

### Nucleotide substitution rates

The *C*. *pauciovulata* and *N. nucifera* plastomes shared 67 plastid-encoded and 41 mitochondrial-encoded genes. To examine the rate variation in 108 organellar genes from *C*. *pauciovulata*, nonsynonymous (*d*_N_) and synonymous (*d*_S_) substitution rates were estimated and compared to those of *N. nucifera* (Figure S9). An examination of the rate variation in individual organelle genes revealed gene-specific acceleration in *C*. *pauciovulata.* The mitochondrial-encoded *nad6* and the plastid-encoded *atpE*, *clpP*, *petD*, *petG*, *petL*, *petN*, *rpl20*, *rpl23*, *rpl32*, *rps15*, *rps16*, *ycf1*, *ycf2*, and *ycf4* genes showed high levels of sequence divergence compared to the patterns of nucleotide substitutions in *N. nucifera*. Among them, the *d*_N_/*d*_S_ values for the plastid-encoded *clpP* gene of *C*. *pauciovulata* were greater than one.

**Fig. 7:**
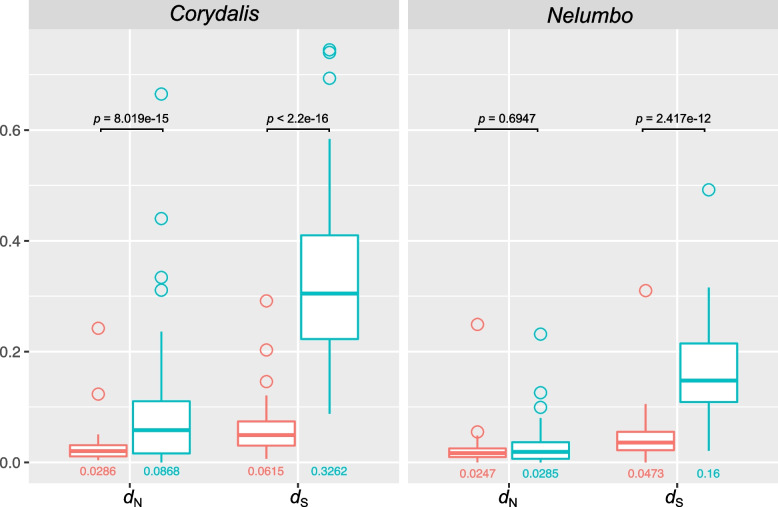
Boxplots of *d*_N_ and *d*_S_ values for plastid and mitochondrial genes in *Corydalis pauciovulata* and *Nelumbo nucifera*. The box represents values between quartiles, the solid lines extend to the minimum and maximum values, and the horizontal lines in the boxes show the median values. The numbers below the boxes represent the mean values

The estimates of nucleotide substitution rates in *C*. *pauciovulata* organelle genomes showed that plastid genes evolved significantly faster than mitochondrial genes in terms of *d*_N_ and *d*_S_ (*C*. *pauciovulata*, *d*_N_: 3.05-fold, *d*_S_: 5.3-fold; Fig. 7). The mitochondrial rates of *C*. *pauciovulata* were very similar to that of *N. nucifera* (*d*_N_: 1.16-fold, *d*_S_: 1.3-fold; Fig. 7). However, the plastid rates of *C*. *pauciovulata* were 2.11 times greater for *d*_N_ and 2.04 times greater for *d*_S_ than for *N. nucifera* (Fig. 7).

**Fig. 8:**
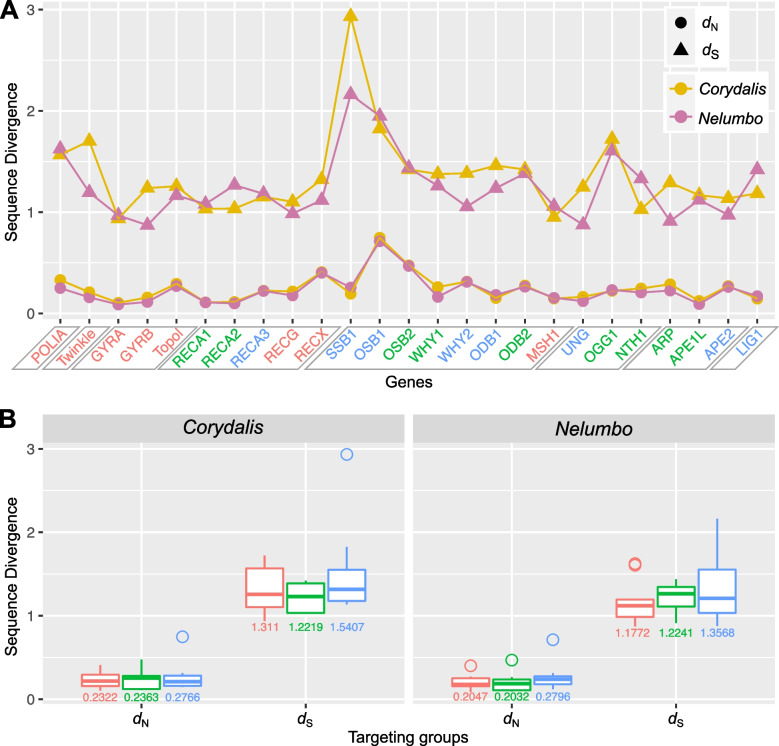
Sequence divergence of 25 DNA replication, recombination, and repair system genes. **A** Nonsynonymous (*d*_N_) and synonymous (*d*_S_) divergence values for 25 individual genes are plotted for *C*. *pauciovulata* and *N. nucifera.* Dual-targeted, plastid-targeted, and mitochondrial-targeted genes are indicated in red, green, and blue, respectively. The DNA-RRR genes are grouped into nine categories by gray parallelograms. **B** Boxplots of *d*_N_ and *d*_S_ values for the target groups. The box represents values between quartiles, the solid lines extend to the minimum and maximum values, and the horizontal lines in the boxes show the median values. The numbers below the boxes represent the mean values. The colors corresponding to the target groups (red, dual-targeted; green, plastid-targeted; and blue, mitochondrial-targeted genes)

To investigate the differences between DNA-RRR genes from *C*. *pauciovulata* and *N.* *nuicifera*, substitution rates were calculated. Among the 25 nuclear-encoded genes, many *C*. *pauciovulata* genes (except *RECA1*, *SSB1*, *ODB1*, *MSH1*, *OGG1*, and *LIG1*) had slightly greater *d*_N_ values than those of *N. nucifera* (Fig. 8A). The *Twinkle*, *GYRB*, *Topol*, *RECG*, *RECX*, *SSB1*, *WHY1*, *WHY2*, *ODB1*, *ODB2*, *UNG*, *OGG1*, *ARP*, *APE1L*, and *APE2* genes from *C*. *pauciovulata* had relatively high *d*_S_ values (Fig. 8A). In the *C*. *pauciovulata* comparison of *d*_N_ and *d*_S_ among the nuclear-encoded genes, there was no significant difference between the targeted groups (Fig. 8B).

## Discussion

In plant cells, organelle genomes require the import of nuclear-encoded organelle-targeted proteins involved in organelle genome stability, including DNA-RRR proteins [7, 8], due to endosymbiotic gene transfers [3, 30]. Modification of DNA-RRR genes is a potential cause of genome complexity [14, 30, 31]. To fully explore the correlations between the modifications of DNA-RRR genes and organelle genome complexity, it is important to produce a high-quality reference genome. However, it is challenging to assemble plastid and mitochondrial genomes that harbor repeats longer than the read length of a single-type platform for short reads [32]. Long reads generated by the ONT or PacBio platform can improve the accuracy and reliability of organelle genome structure compared with those generated by a short-read-based assembly [33, 34].

### Structural variations in *Corydalis pauciovulata* organelle genomes

In this study, we generated high-quality assemblies of the complete plastid and mitochondrial genomes of *C*. *pauciovulata* by combining two different Illumina libraries (one paired end and one mate pair) and ONT reads. In addition, we identified 25 DNA-RRR genes from *C. pauciovulata* and estimated substitution rate variations in each DNA-RRR gene, and the findings provide numerous opportunities for research on organelle genome stability in the family Papaveraceae. We have shown that the *C*. *pauciovulata* plastome has undergone dynamic changes that distinguish it from most angiosperm plastomes, similar to the findings for other members of the same genus. Many *Corydalis* species have been identified as having rearranged plastomes, including IR expansions and gene losses [24, 27, 28]. Our plastome showed conflicting structures and sizes with those of the two published plastomes of *C. pauciovulata* (MK264352; 161,773 bp and NC_072192; 159,167 bp), although we cannot rule out the possibility of heterogeneous divergence in the plastomes of *C*. *pauciovulata*. For example, our plastome contained an expanded IR (46,060 bp), whereas the two published plastomes contained IRs of typical sizes (MK264352; 22,719 bp and NC_072192; 22,777 bp). Nucleotide sequence alignment of three plastomes with one IR showed that our plastome was highly similar to that of MK264352 with 98.6% identity, whereas NC_072192 was divergent, with 91.9% identity, from our plastome. These conflicting findings are difficult to interpret because of the lack of a detailed assembly method, and whether the plastome was generated using a reference or de novo approach has not been reported. Notably, the two published plastomes were generated using only short reads and different assembly tools, which may have contributed to the observed differences in plastome structure and size compared with our assembly. In plant mitogenomes, recombination with repeats results in multiple isomeric master and subgenomic circles. The *C. pauciovulata* mitogenome exhibits a dynamic genome structure that can be shaped by intramolecular recombination, and we demonstrated that homologous recombination is associated with five repeats, resulting in multiple isomeric master circles (Fig. 8). However, additional configurations, including subgenomic circles, may be present in the mitochondria of *C. pauciovulata*. Recombination activity in the mitogenome can disrupt conserved gene clusters. Comparative gene cluster analysis showed that the *C. pauciovulata* mitogenome may have undergone more rearrangements than the *N. nuicifera* mitogenome because two additional more missing gene clusters were inferred for *C. pauciovulata*.

### Evolutionary dynamics of organelle genes

A comprehensive comparison of the nuclear and organelle genomes could help identify fates or factors that impact the evolution of mitogenomes and plastomes, including gene losses, rearrangements, and accelerated substitution rates. Although the loss of several plastid-encoded genes in *Corydalis* plastomes has been documented [24, 25, 27, 28], the evolutionary fates of these genes are unclear. Our results showed that the *C. pauciovulata* plastome lacked 12 protein-coding genes (*accD* and 11 *ndh* genes), but the mitogenome contained 41 protein-coding genes that are ancestral in angiosperms. The plastid *accD* gene was independently lost during angiosperm evolution, and two mechanisms of functional replacement to the nucleus have been documented for *accD*: 1) IGT in some Geraniaceae [35] and *Trifolium* [36, 37] and 2) gene substitution by a cytosolic homolog of eukaryotic origin in Brassicaceae [38, 39], Geraniaceae except for *Hypseocharis* [35], and Poaceae [40, 41]. Nuclear genome and transcriptome data revealed that IGT of *accD* from plastids to the nucleus occurred in *C*. *pauciovulata* instead of as a gene substitution, and the nuclear-encoded *ACCD* gene acquired an intron (Fig. 6). A previous study showed that the loss of *accD* occurred in the common ancestor of *Corydalis* [28]. Taken together, these results suggest a single ancient transfer from plastids to the nucleus in this lineage.

In contrast to *accD*, there is no evidence of functional replacement of the plastid-encoded *ndh* genes in the nucleus, although the *ndh* complex plays a role in electron transport during photosynthesis [42]. A suite of nuclear-encoded NDH-related protein complexes that assemble plastid-localized *ndh* genes is required for photosynthesis [43]. The parallel loss of NDH-related protein complexes from nuclear and plastid genomes has been reported [44, 45]. These results suggest that the plastid NDH complex has been lost in cells or that it has been functionally replaced by alternative factors. The loss of plastid *ndh* genes has been observed not only in parasitic [46–48], mycoheterotrophic [45], and carnivorous plants [49, 50] but also in multiple photoautotrophic lineages [51–56]. Multiple losses or degradations of plastid *ndh* genes have occurred during *Corydalis* plastome evolution [24, 25, 27, 28]. Although it is still unclear which factors contribute to the loss of the plastid *ndh* gene, possible explanations for this loss have been suggested through evolutionary adaptation during the transition to heterotrophic lifestyles [45, 57] or arid conditions. In the *C. pauciovulata* transcriptome, we also detected no transcripts of the nuclear-encoded *ndh* gene for plastids and only a few nuclear-encoded NDH-related genes, suggesting potential losses in *C. pauciovulata*.

The *clpP* gene was previously annotated as a pseudogene or was lost [27, 28]; however, we found that all the sequenced *Corydalis,* including our *C. pauciovulata* plastome, contained the *clpP* gene in their plastomes. The *clpP*, encoded by plastids, is crucial in protein metabolism, functioning in the degradation and turnover of damaged or misfolded proteins within the organelle [58, 59]. This gene typically contains two introns, which are conserved across many plant lineages. In some cases, angiosperm lineages have been found to lack one or both introns within the *clpP* gene, revealing a correlation between increased substitution rates and structural changes in *clpP* genes [35]. The plastid-encoded *clpP* gene of *C. pauciovulata* exhibited *d*_N_/*d*_S_ values greater than one, but its characteristic structure contained two introns. The increased substitution rates and the presence of introns in *clpP* genes observed in *C. pauciovulata* raise intriguing questions about the evolutionary dynamics of this gene. The identification of a large insertion in the first exon of the *clpP* gene of *C. pauciovulata* adds to our knowledge of plastid genome diversity and structural variation within this species. However, further investigation are needed to assess the functional consequences of any structural alterations, such as the large insertion in the first exon, and whether they impact the functionality of the gene.

### Impact of DNA replication, recombination, and repair genes on organelle genome stability in *Corydalis pauciovulata*

Angiosperm organelles do not encode genes associated with the DNA repair system; thus, DNA-RRR genes must be imported into plastids or mitochondria to maintain the organelle genome stability [7]. The modification of DNA-RRR genes has also been proposed to drive genome rearrangements and rate accelerations in the organelle genomes of angiosperms [15]. We also suggest that the dynamic structure of the *C. pauciovulata* plastome may result from mutations in some of the DNA-RRR genes. Our analyses showed that some specific DNA-RRR genes of *C. pauciovulata*, which target mitochondria (*WHY2*, *ODB1*, and *UNG*), plastids (*WHY1*, *ODB2*, *ARP*, and *APE1L*), and both (*Twinkle*, *GYRB*, *Topol*, *RECG*, and *RECX*), had higher *d*_N_ and *d*_S_ values than those of *N. nuicifera*. An increase was found for the *d*_N_ and *d*_S_ of dual-targeted, *d*_N_ of plastid-targeted, and *d*_S_ of mitochondrial-targeted gene groups relative to those in *N. nuicifera*. Previous studies revealed that plastid-targeted *WHY1* and dual-targeted *RECG* and *MSH1* proteins help maintain plastid genome stability by preventing illegitimate recombination [30, 31, 60], showing that knockouts or high mutation rates of these genes increase the frequency of recombination in both mitochondria and plastids. However, *MSH1* in *C. pauciovulata* displayed lower *d*_N_ and *d*_S_ values than that in *N. nuicifera*. *MSH1* affects the genomes of both organelles; therefore, additional mitochondrial genome sequences are needed to explain this phenomenon. To better address the fundamental question about the correlation between the modification of DNA-RRR genes and organelle genome stability, additional genomic resources from other members of the family Papaveraceae are needed to examine distinct patterns of sequence divergence between the conserved and dynamic genome groups.

## Conclusions

Our results provide a valuable resource for better understanding the evolution of *Corydalis* organelle genomes. In particular, the first mitogenome of Papaveraceae provides an example that other researchers can explore by sequencing the mitogenomes of related plants. Mutation or dysfunction of DNA-RRR systems has been hypothesized to cause plant organelle genome instability [7]. Our results provide fundamental information about DNA-RRR genes in *Corydalis* and their related rate variation, shedding light on the relationships between DNA-RRR genes and organelle genome stability. This highlights the importance of further research to elucidate the mechanistic underpinnings of DNA-RRR function and its impact on the evolutionary trajectories of organelle genomes across plant lineages.

Future research could focus on investigating the specific mechanisms by which DNA-RRR systems influence organelle genome stability in *Corydalis* and related taxa. In addition, comparative studies across a broader range of Papaveraceae species could provide valuable insights into the evolutionary conservation or divergence of DNA-RRR gene function and its implications for plant adaptation and diversification.

## Methods

### DNA extraction and genome sequencing

*Corydalis pauciovulata* individual was collected from Mt. Bohyeon in Yeongcheon-si, South Korea [voucher *Seongjun Park 2018* (YNUH)]. Total genomic DNA (11.4 μg) was extracted from fresh leaves using the DNeasy Plant Mini Kit (Qiagen, Hilden, Germany) following the manufacturer’s protocol. The *Corydalis* DNA was sequenced using the Illumina HiSeq2000 platform (Illumina, San Diego, CA, USA) with two libraries: 100 bp × 2 paired-end (PE) reads from a 550 bp library and 100 bp × 2 mate-pair (MP) reads from a 3,000 bp library. In addition, long reads were generated using the Oxford Nanopore Technologies (ONT) GridION platform (ONT, Oxford, United Kingdom).

### Organelle genome assemblies and annotation

The organelle genomes of *C. pauciovulata* were assembled using three approaches: 1) A standard method using Illumina PE reads, 2) a combined method using the Illumina PE and MP reads, and 3) a hybrid method using both Illumina and ONT data. For the standard and combined methods, Velvet v1.2.10 [61] was used to assemble the genomes with multiple *k*-mers (69 to 95) and expected coverage values (100, 200, 300, 400, 500, and 1000). For the hybrid method, SPAdes v3.13.1 [62] was used with multiple cutoff (0, 25, 50, 100, 200, and 300) values and the “careful” option. The de novo organelle genome assemblies were performed on a 32-core 3.33 GHz Linux workstation with 512 GB of memory. Circular plastid and mitochondrial genomes were assembled in Geneious Prime 2021.1.1 (www.geneious.com) by mapping contigs onto the longest contigs and merging, and the overcollapsed contigs were used to infer boundaries of repeat regions. To assess the depth of coverage for the completed genomes, Illumina PE/MP reads were mapped to the whole plastome and mitogenome sequences with Bowtie v2.2.9 [63], and ONT reads were mapped to the genomes with BWA v0.7.17 [64]. The *C*. *pauciovulata* plastid and mitochondrial genomes were annotated using a BLAST-like algorithm (50% similarity) in Geneious Prime with the protein-coding genes from *Liriodendron tulipifera* organelle genomes (NC_008326 and NC_021152), and their open reading frame (ORF) was confirmed using “Find ORFs” in Geneious Prime. All tRNA genes in the organelle genomes were predicted using tRNAscan-SE v2.0.9 [65] and ARAGORN v1.2.38 [66]. Circular organelle genomes were drawn with OGDRAW v1.3.1 [67]. The genomes were deposited in GenBank (accession numbers OR100521 and OR100522).

### Comparative analyses

Dispersed repeat sequences in organelle genomes were identified by performing “BLASTN” searches against themselves using BLAST + v2.12.0 [68], with a word size of 16 and an *e*-value of 1 × 10^–6^. Mitochondrial DNAs of plastid origin (MIPTs) were identified by performing “BLASTN” searches of the *C. pauciovulata* plastome against its mitogenome with an *e*-value cutoff of 1 × 10^–6^, at least 80% sequence identity and a minimum length of 50 bp. Additionally, “BLASTN” searches of all 11 *ndh* and *accD* genes from the *Lamprocapnos spectabilis* plastome (NC_039756) against the *C. pauciovulata* mitogenome were also performed because the 12 plastid genes were lost or pseudogenes in the *C. pauciovulata* plastome. ORFs longer than 150 bp in the mitochondrial genome were predicted using the “Find ORFs” option with the ATG start codon in Geneious Prime. Any ORFs that overlapped with the annotated mitochondrial genes and MIPTs were excluded. To identify a conserved domain (CD), ORFs were translated, and CD searches were performed against the Conserved Domain Database (CDD) v3.19 [69]. To search for potential CMS-type ORFs in the *C. pauciovulata* mitogenome, all ORFs were compared with the annotated mitochondrial genes using “BLASTN” with an *e*-value cutoff of 1e-3, a minimum length of 30 bp, and at least 90% sequence identity. The TMHMM Server v.2.0 [70] was used to predict transmembrane helices in selected ORFs. Forty-one mitochondrial genes were searched using PREP-Mt [71] with a cutoff value of 0.5 to predict RNA editing sites. The available mitochondrial transcripts in the *C*. *pauciovulata* transcriptome (see below) were identified using BLAST + and aligned with the genomic gene sequences to verify the empirical RNA editing sites on the protein-coding genes. In addition, we mapped the corrected reads (see below) to the genomic gene sequences using Bowtie2 to confirm the sites.

### Identification of organelle-targeted genes in the nucleus

Total RNA was isolated from fresh leaves using the methods of Breitler et al. [72]. The *Corydalis* RNA was sequenced using the Illumina HiSeq2000 platform with PE reads, and error correction for the PE reads was performed using Rcorrector v1.0.4 [73]. To identify organelle-targeted genes in the nucleus, transcriptomes from *C*. *pauciovulata* were assembled de novo using Trinity v2.13.2 [74] with the “trimmomatic” option. The transcripts were examined for completeness of the assembly using Benchmarking Universal Single-Copy Orthologs (BUSCO) v5.2.2 [75] with the lineage “eudicots_odb10”. The IGT events were identified using “BLASTN” (*e*-value cutoff of 1e-10) of the 41 mitochondrial-encoded genes of the *L. tulipifera* mitogenome and the 79 plastid-encoded genes of the *L. spectabilis* plastome as queries. Four plastid-encoded genes, *accD*, *rpl20*, *rpl23,* and *rps16,* have been substituted by a cytosolic homolog of an eukaryotic or mitochondrial origin [35, 38–41, 76–82]. To investigate the possible substitution of these genes in *C*. *pauciovulata*, the amino acid sequences of nuclear eukaryotic acetyl-CoA carboxylase (*ACC*) (AT1G36180 from *Arabidopsis thaliana*), *RPL20* (AT1G16740 from *A. thaliana*), *RPL23* (Q9LWB5 from *Spinacia oleracea*), and *RPS16* (AB365526 from *Medicago truncatula*) were used to perform a “BLASTP” (*e*-value cutoff of 1e-6) search against the translated *Corydalis* transcriptome. To detect the nuclear-encoded NDH complex in the nucleus, protein sequences from *Arabidopsis thaliana* were downloaded from The Arabidopsis Information Resource (TAIR) [https://www.arabidopsis.org/] as references. The reference protein sequences were aligned to the *Aquilegia coerulea* v3.1 transcriptome from the genomics portal Phytozome v12.1.6 (https://phytozome.jgi.doe.gov/pz/portal.html) using “BLASTP” to extract the nuclear-encoded NDH complex of *A. coerulea* (Table S5). The protein sequences from both *A. thaliana* and *A. coerulea* were used as queries against the translated *Corydalis* transcriptome. The chloroplast transit peptide (cTP), mitochondrial targeting peptide (mTP) and its cleavage site were predicted using TargetP v1.1 [83].

For the DNA-RRR genes, we focused on 32 nuclear genes from *A. thaliana* that were found to target plastids, mitochondria, or both [84] and used them as queries for “BLASTP” searches against the translated *C. pauciovulata* transcriptome (Table S6). Transcriptomes from *L. tulipifera* (SRR8298316) and *N. nucifera* (SRR8298325) were also assembled de novo using the Sequence Read Archive (SRA) with Trinity to retrieve the DNA-RRR gene sequences.

### Estimation of structure and substitution rate variation

The *C*. *pauciovulata* organelle genomes were aligned with the published *N. nucifera* plastid (KM655836) and mitochondrial (NC_030753) genomes from Proteales, which are available for comparison based on complete organelle genomes, using the “progressiveMauve” algorithm in Mauve v2.3.1 [85] in Geneious Prime. Organelle genomes from *L. tulipifera* were used as a reference. The nonsynonymous (*d*_N_) and synonymous substitution (*d*_S_) rates of organelle genes from *C*. *pauciovulata* and *N. nucifera* were calculated in KaKs_calculator v2.0 [86], employing the GY-HKY substitution model. Protein-coding genes from the *L. tulipifera* organelle genomes were used as a reference. Individual protein-coding genes were aligned based on the back-translation approach with MAFFT v7.017 [87] in Geneious Prime. Statistical analyses were conducted with R v4.1.2 [88].

The *d*_N_ and *d*_S_ rates of DNA-RRR genes from *C*. *pauciovulata* and *N. nucifera* were also calculated as described above. DNA-RRR genes from the *L. tulipifera* transcriptome were also used as a reference. To identify introns and exons in the DNA-RRR genes, a draft nuclear genome for *C*. *pauciovulata* was assembled using MaSuRCA v4.0.1 [89]. Nucleotide sequences of DNA-RRR genes from *C*. *pauciovulata* were used as queries against the draft genome of *C*. *pauciovulata* and aligned with identified nuclear contigs using MUSCLE [90] to determine the intron/exon boundaries.

### Supplementary Information

**Supplementary Material 1.**

## Data Availability

The data sets supporting the results of this article are included in additional files. Complete mitochondrial and plastid genome sequences are available in GenBank (https://www.ncbi.nlm.nih.gov/nuccore/OR100521, OR100522).

## Abbreviations

*d*_N_: Number of substitutions per nonsynonymous site
*d*_S_: Number of substitutions per synonymous site
LSC: Large single copy
SSC: Small single copy
IR: Inverted repeat
IGT: Intracellular gene transfer
MIPTs: Mitochondrial DNAs of plastid origin
PLMTs: Plastid DNAs of mitochondrial origin
DNA-RRR: DNA replication, recombination, and repair
