# Supplementary material for: Dynamic changes in the plastid and mitochondrial genomes of the angiosperm Corydalis pauciovulata (Papaveraceae)
Source: BMC Plant Biol. 2024 Apr 22;24:303. doi: 10.1186/s12870-024-05025-4 (PMC11034061; doi:10.1186/s12870-024-05025-4)
Supplement: Supplementary file 1 — Supplementary Material 1. [file 12870_2024_5025_MOESM1_ESM.pdf]

Article title: Dynamic changes in the plastid and mitochondrial genomes of the angiosperm *Corydalis pauciovulata* (Papaveraceae)

Authors: Seongjun Park, Boram An, SeonJoo Park

Additional material

Figure S1. Sequencing coverage of *the Corydalis pauciovulata* plastome and mitogenome.

Figure S2. Structural alignments of the *Corydalis pauciovulata* plastome with outgroups.

Figure S3. Illustration of corrected ONT reads mapped against the assembled *Corydalis* mitogenome.

Figure S4. Mitochondrial maps surrounding the *orf146*, *orf244*, and *orf457ab.*

Figure S5. BUSCO assessment of the three transcriptomes.

Figure S6. Characterization of the nuclear-encoded acetyl-CoA carboxylase beta subunit (*ACCD*) genes of *Corydalis pauciovulata* .

Figure S7. Characterization of the nuclear-encoded *ACC*, *RPL20*, *RPL23*, and *RPS16* genes of *Corydalis pauciovulata*.

Figure S8. Genome completeness size and estimation.

Figure S9. Variation in sequence divergence among in *Corydalis pauciovulata* and *Nelumbo nucifera* plastid and mitochondrial protein-coding genes.

Table S1. List of annotated genes in the *Corydalis pauciovulata* plastome.

Table S2. Blast result of plastid-derived DNA segments in the *Corydalis pauciovulata* mitogenome.

Table S3. Predicted RNA editing in 38 protein-coding genes for the *Corydalis pauciovulata* mitogenome.

Table S4. RNA editing validation of 21 genes using transcriptome data.

Table S5. Blast results of ORFs (>150 bp) in the *Corydalis pauciovulata* mitogenome.

Table S6. Potential chimeric ORFs.

Table S7. Information on the nuclear-encoded NDH complex genes.

Table S8. Information on the nuclear-encoded DNA-RRR genes.

Figure S1. Sequencing coverage of *the Corydalis pauciovulata* plastome and mitogenome. Linear plastome (A) and mitogenome (B) maps of the *C. pauciovulata*. The genes above the horizontal line are transcribed in the forward direction; the genes below the horizontal line are transcribed in the reverse direction. Each graph below the maps shows the depth of each sequencing coverages across the *C. pauciovulata* plastome and mitogenome. Red: PE, Green: MP, Blue: ONT.

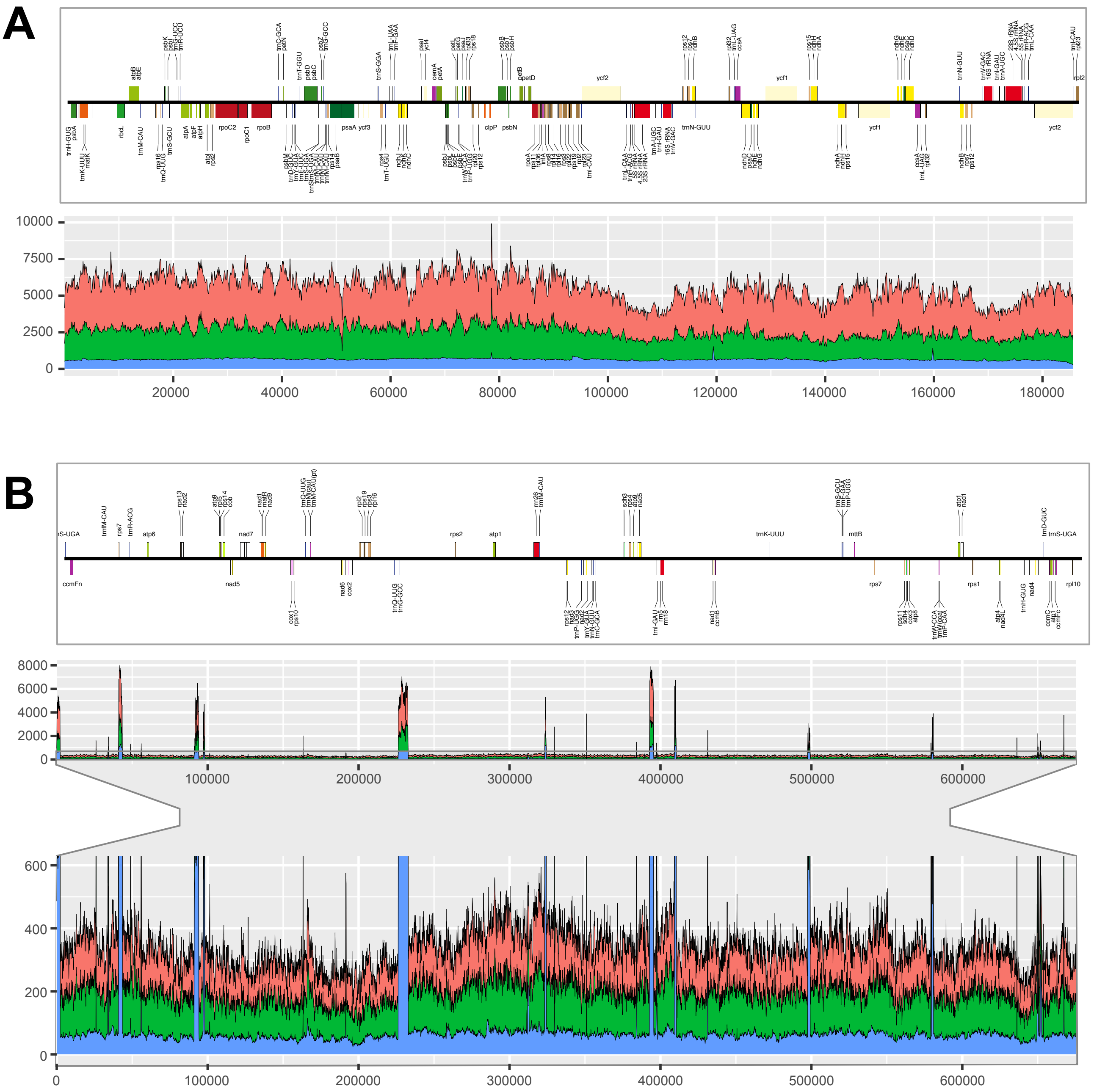

Figure S2. Structural alignments of the *Corydalis pauciovulata* plastome with outgroups. The colored blocks represent collinear sequence blocks shared by all plastomes. Blocks drawn below the horizontal line indicate sequences found in an inverted orientation. Individual genes and strandedness are represented below the *Liriodendron* genome block. Only one copy of the inverted repeat (IR) is shown for each plastome and pink boxes below each plastome block indicate its IR. Red boxes correspond to the purple box (second inversion) on Figure 3.

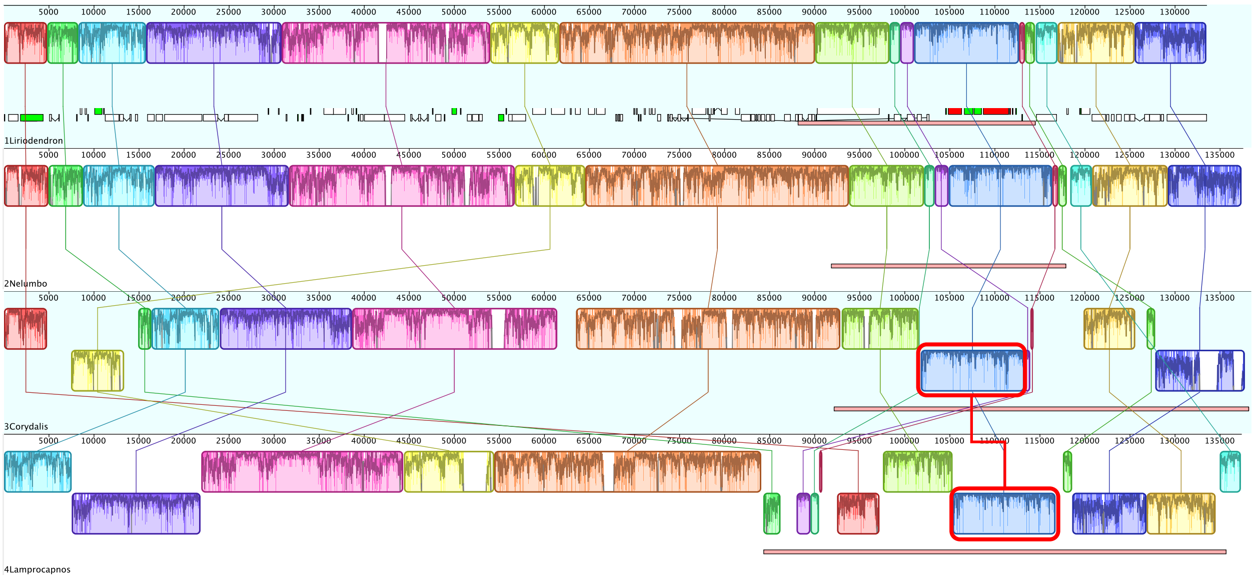

Figure S3. Illustration of corrected ONT reads mapped against the assembled *Corydalis* mitogenome. Blue lines indicate that those regions are identical to the parts of the mitogenome. Red lines indicate regions of ONT reads not aligned to the mitogenome, which are alternative genomic regions.

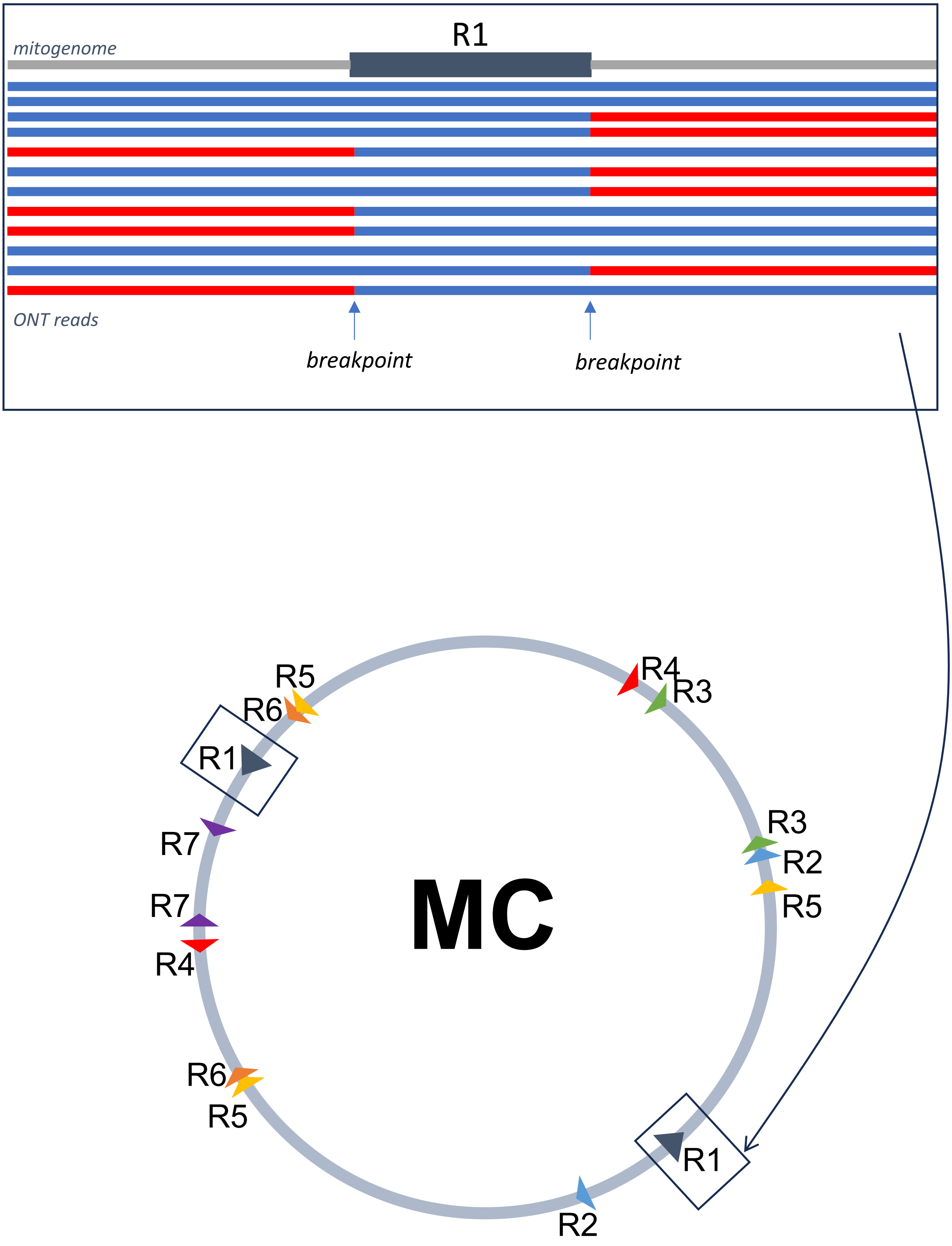

Figure S4. Mitochondrial maps surrounding the *orf146*, *orf244*, and *orf457ab*. Black annotation indicates exons and yellow annotation indicates coding sequence. Green annotation indicates gene. Gray annotation indicates each fragment of the genes. Ocher annotation indicates the repeats (R1, R5, and R6).

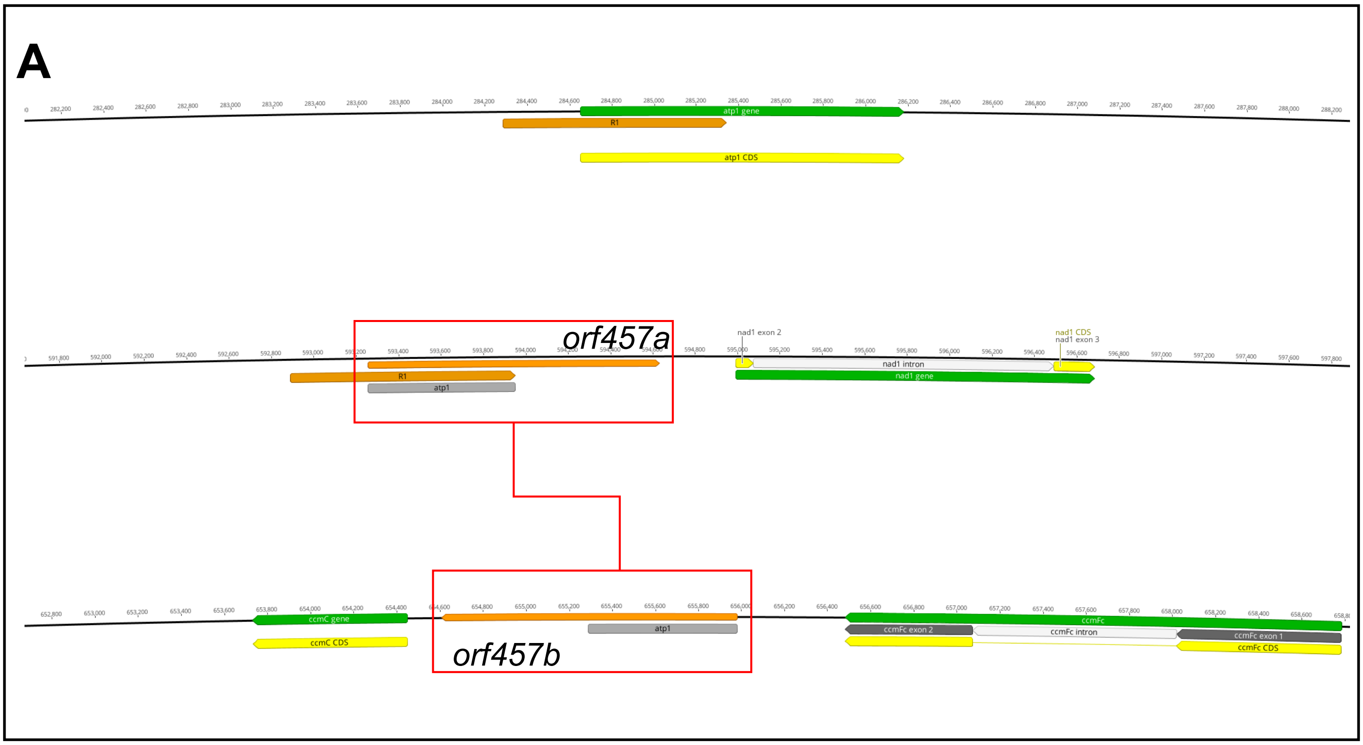

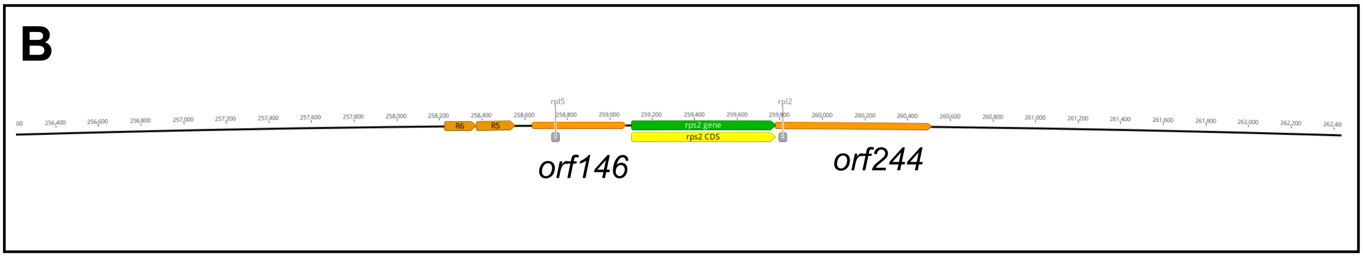

Figure S5. BUSCO assessment of the three transcriptomes. Eudicots database; 2,326 genes.

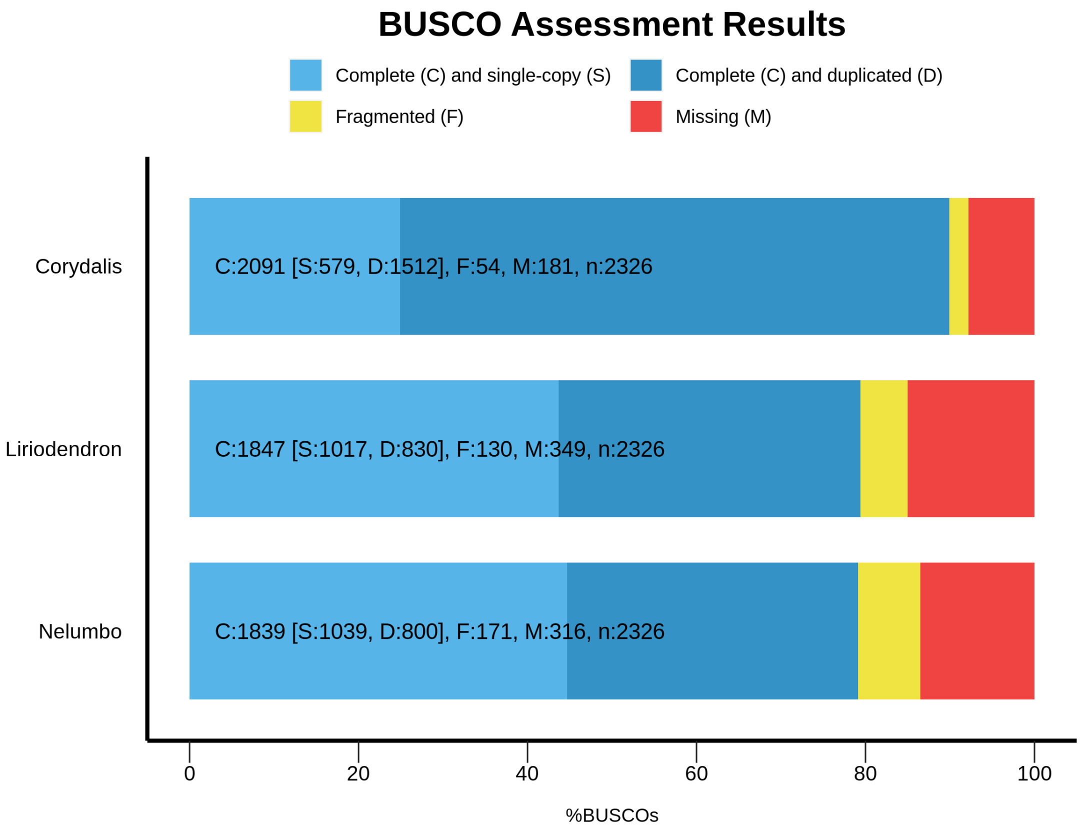

Figure S6. Characterization of the nuclear-encoded acetyl-CoA carboxylase beta subunit (*ACCD*) genes of *Corydalis pauciovulata*. A. Comparison of the genomic DNA and mRNA sequences from *C. pauciovulata ACCD* gene. Pink annotation indicates transit peptide and red annotation indicates a conserved domain of accD. Black annotation indicates exons and yellow annotation indicates coding sequence. Green annotation indicates gene. **B.** Nucleotide sequence alignment of the nuclear and transcript copies of *accD* .

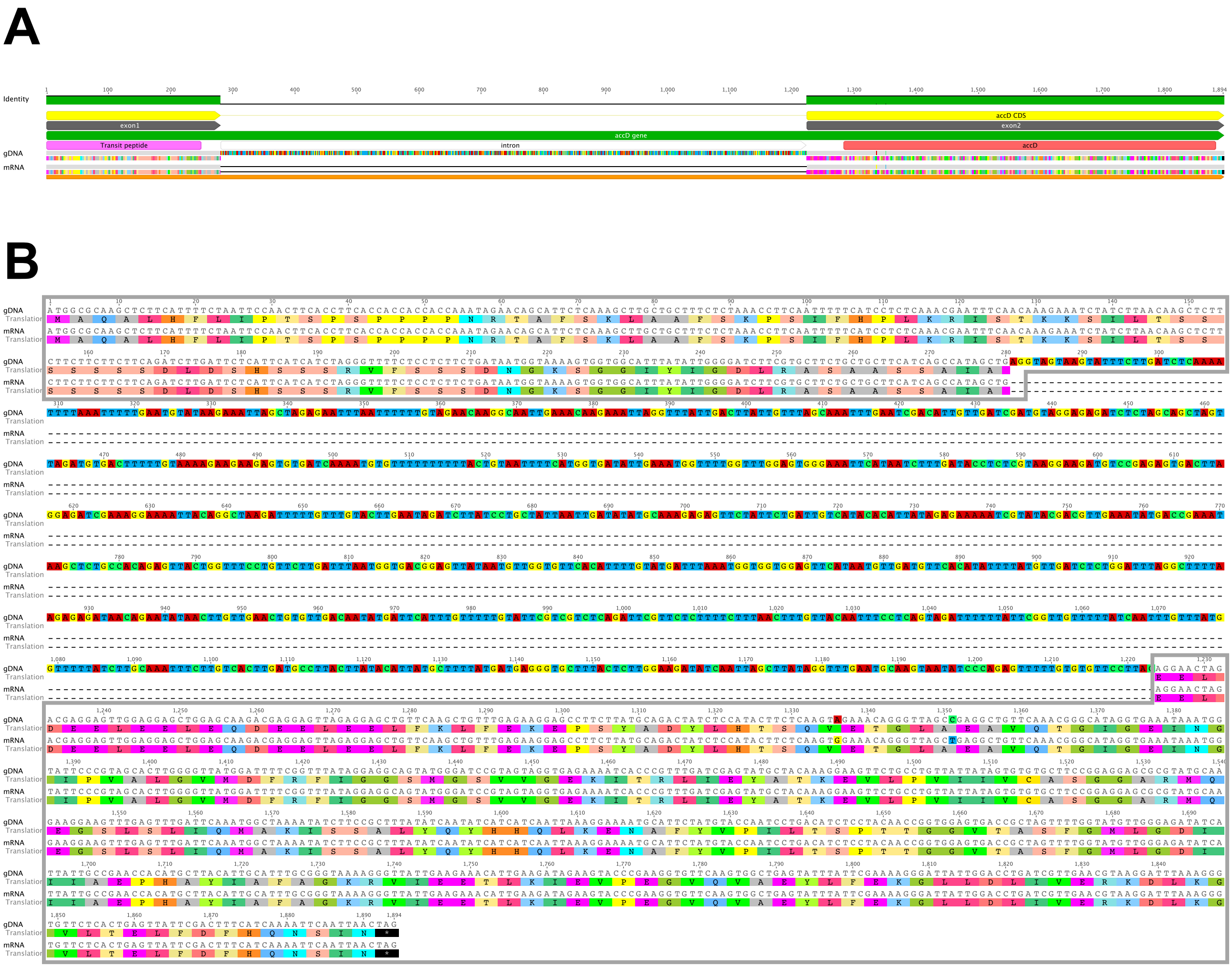

Figure S7. Characterization of the nuclear-encoded *ACC*, *RPL20*, *RPL23*, and *RPS16* genes of *Corydalis pauciovulata*. The nuclear-encoded *RPS16* (a), *ACC (b)* , *RPL20 (c)*, and *RPL23 (d)* genes **A.** Comparison of the genomic DNA and mRNA sequences from *C. pauciovulata*. Pink annotation indicates transit peptide and red annotation indicates a conserved domain of each gene. Black annotation indicates exons and yellow annotation indicates coding sequence. Green annotation indicates gene. **B.** Nucleotide sequence alignment of the nuclear and transcript copies of each gene.

a) *RPS16*

A

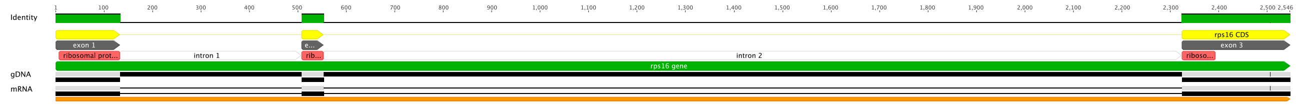

B

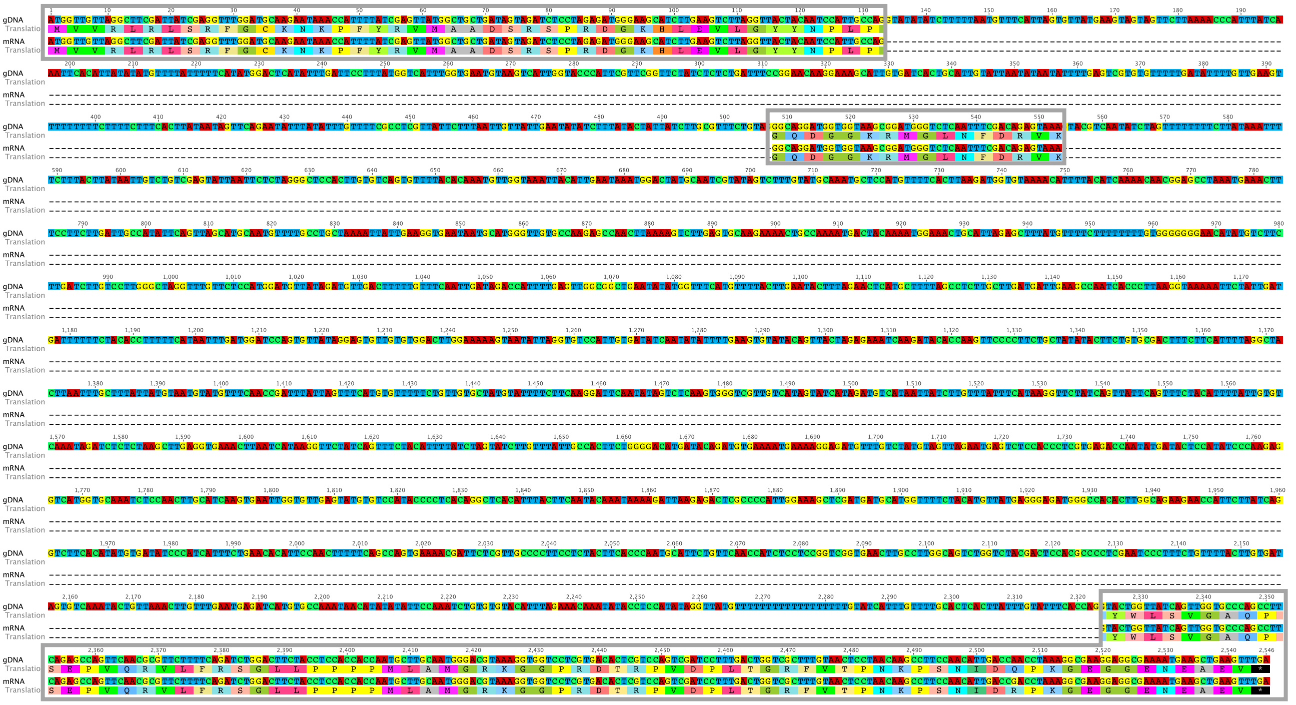

Figure S7. (continued).

b) *ACC*

A

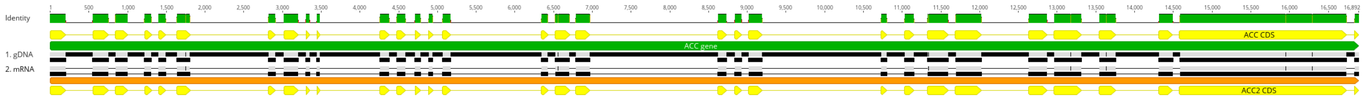

B

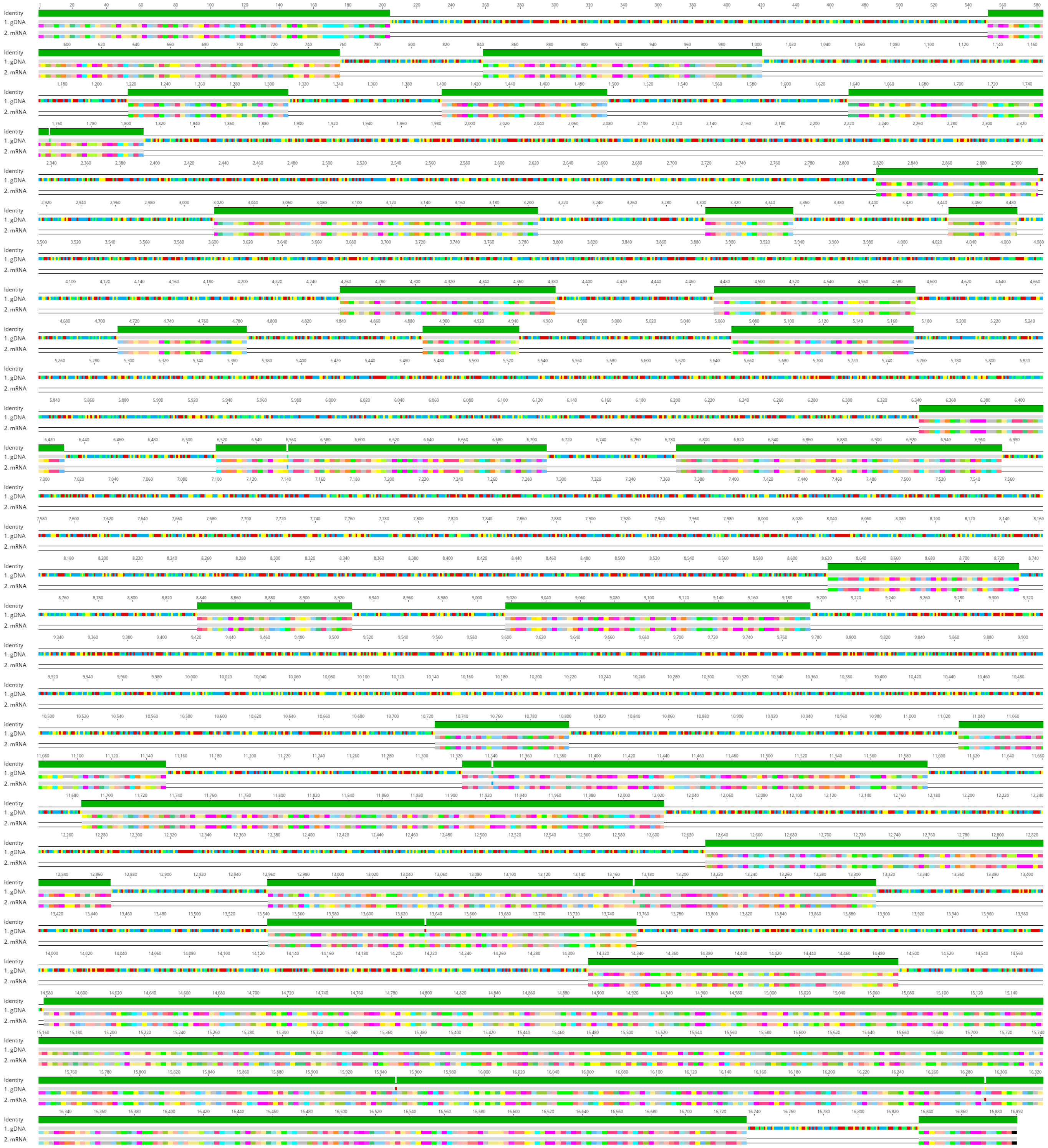

Figure S7. (continued).

c) *RPL20*

A

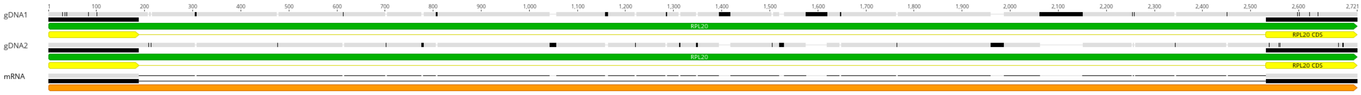

B

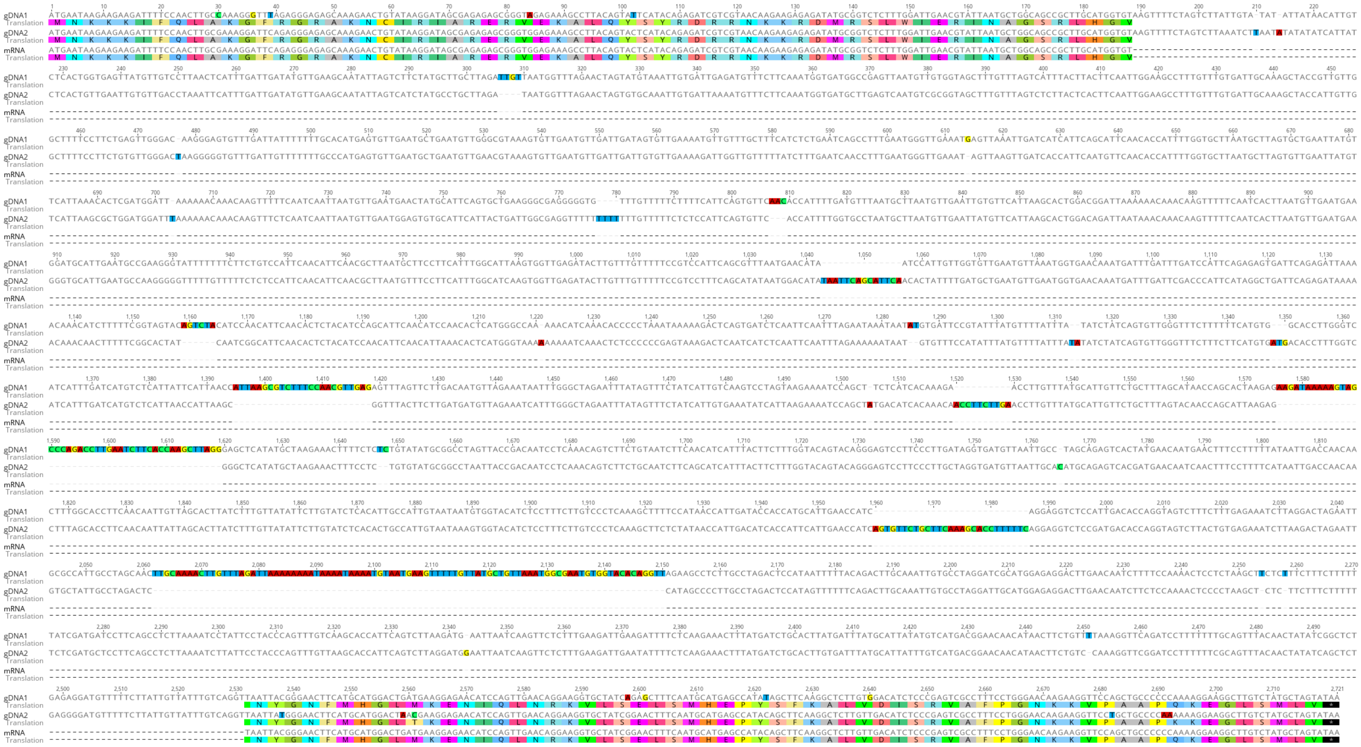

Figure S7. (continued).

d) *RPL23*

A

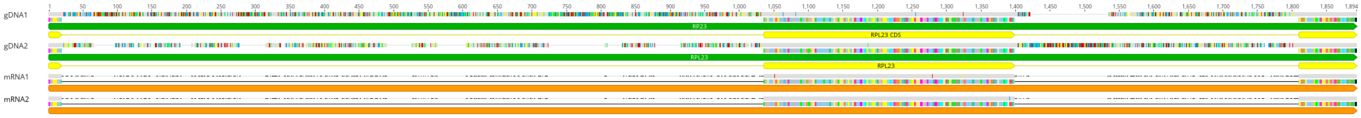

B

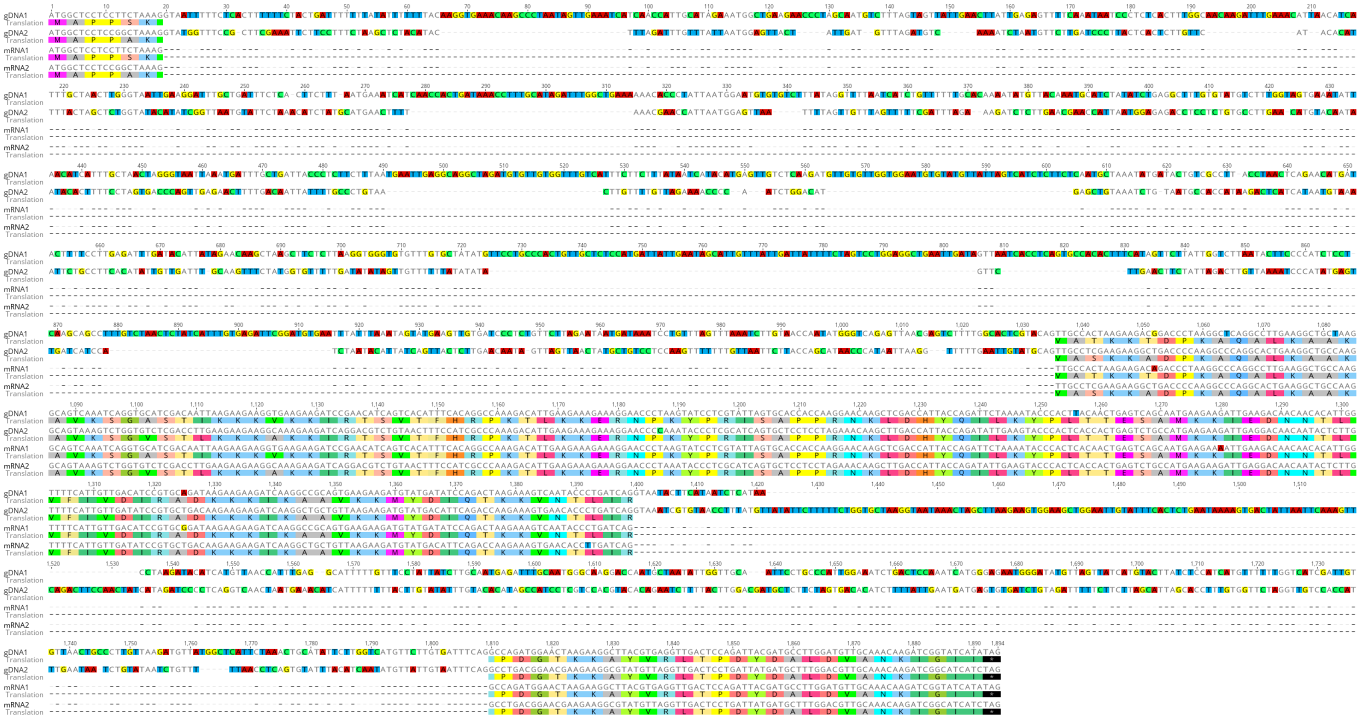

Figure S8. Genome completeness size and estimation. A. GenomeScope plot for *Corydalis pauciovulata.* The 21 *k*-mer distribution used for the estimation of *Corydalis* genome size. **B.** BUSCO assessment of the *Corydalis* genome (eudicots database; 2,326 genes).

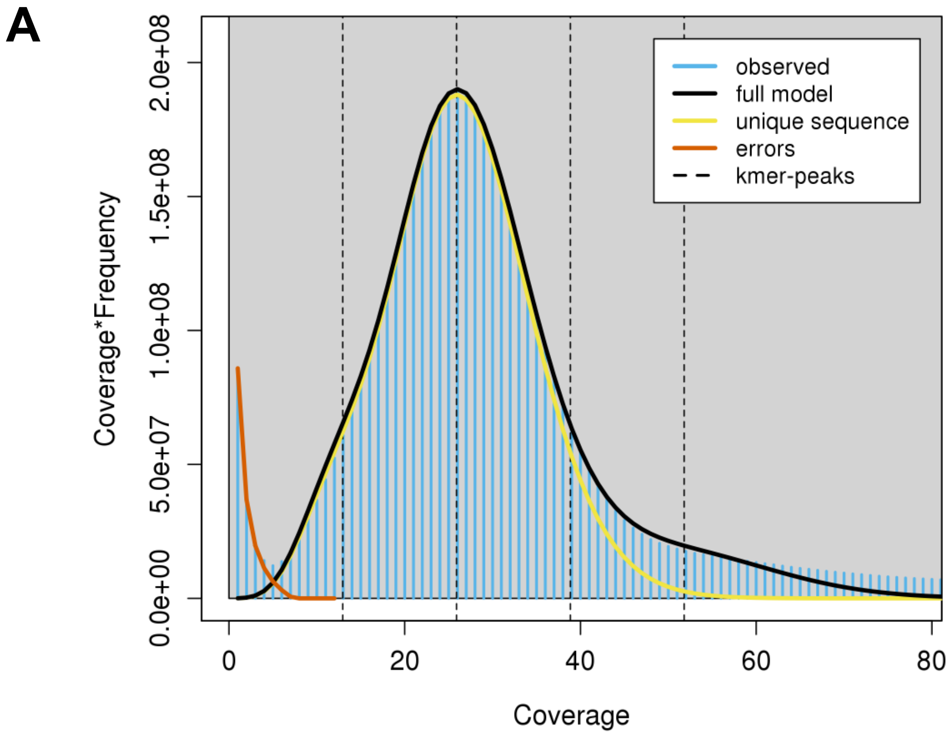

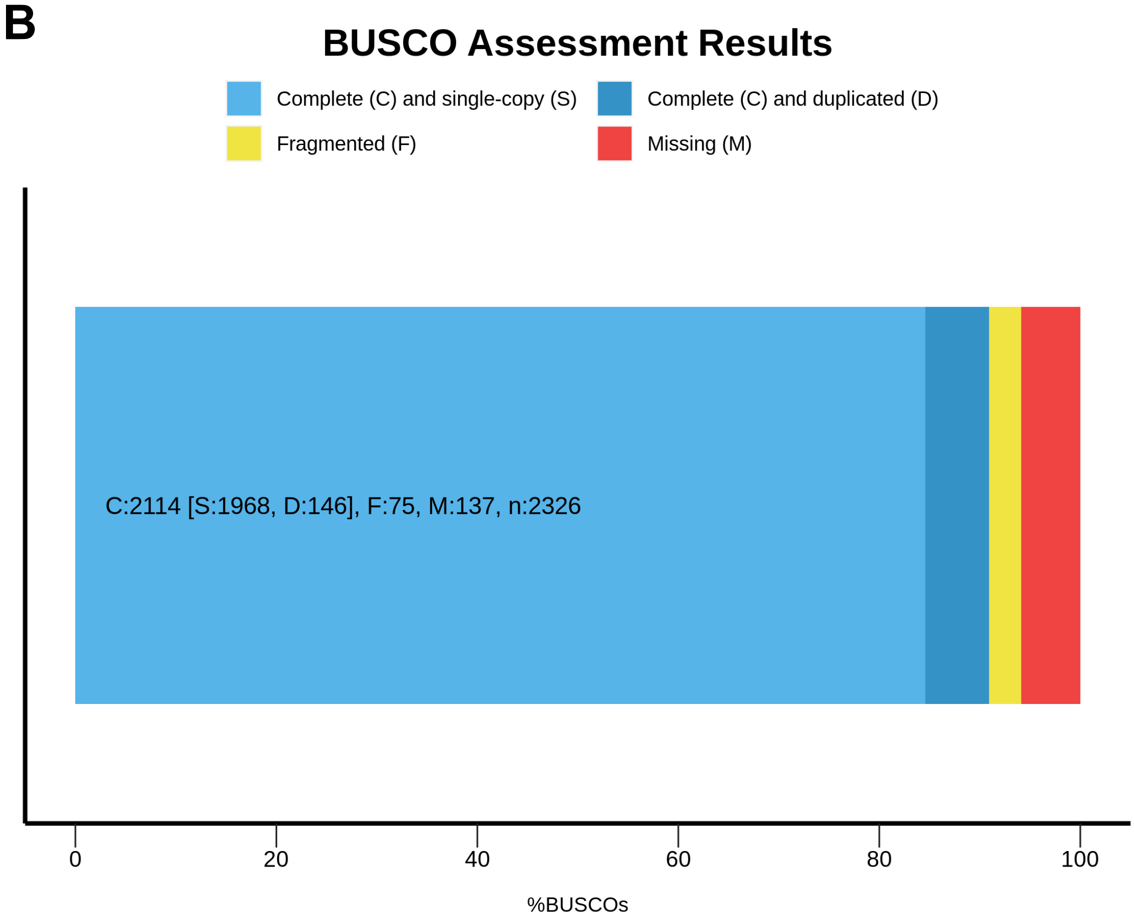

Figure S9. Variation in sequence divergence among in *Corydalis pauciovulata* and *Nelumbo nucifera* plastid and mitochondrial protein-coding genes.

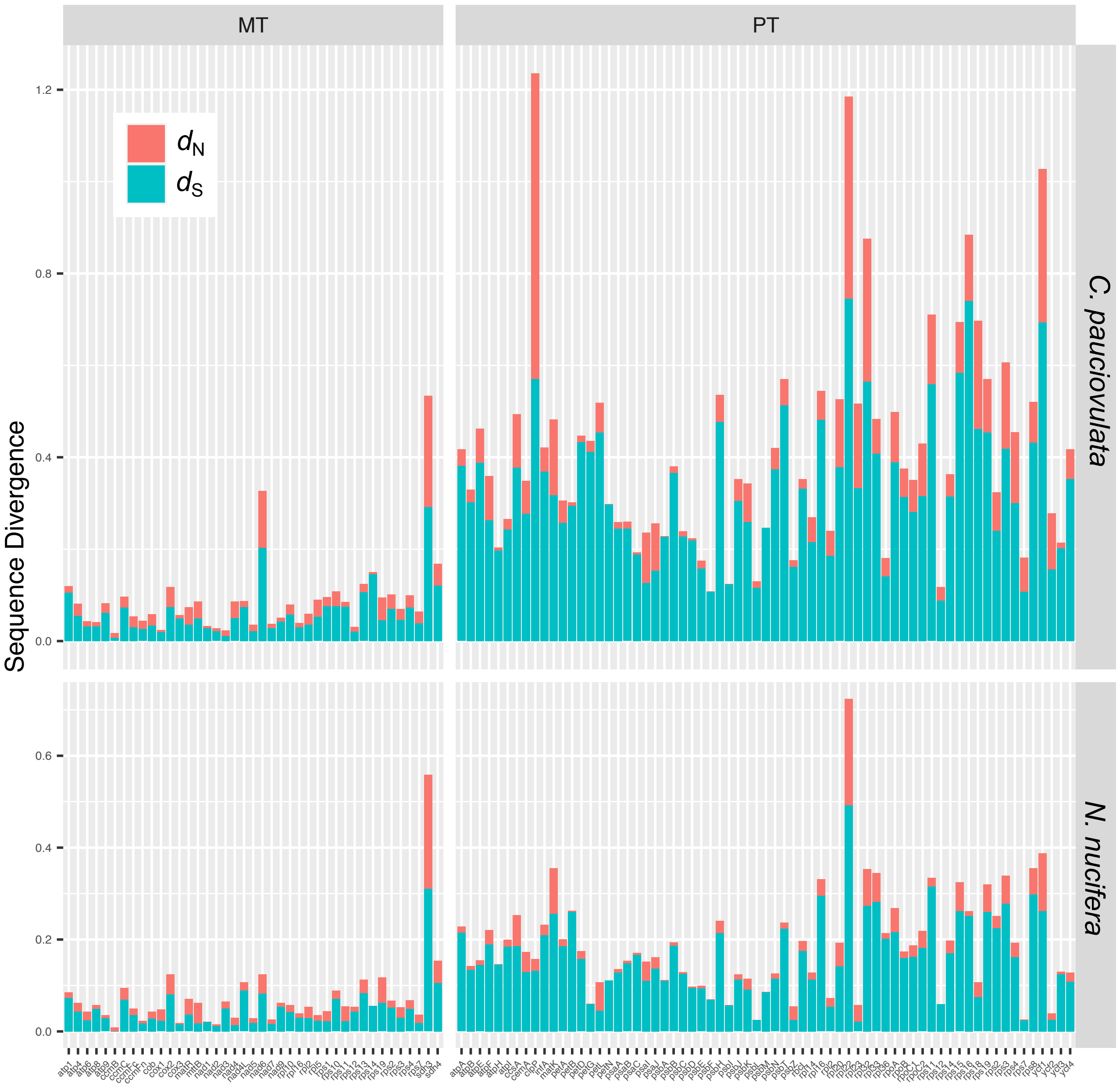

Table S1. List of annotated genes in the *Corydalis pauciovulata* plastome.

Catergory for genes	Group of gene	Name of gene
Genes for photosynthetic apparatus	photosystem Ⅰ	*psaA, psaB, psaC, psaI, psaJ*
	photosystem Ⅱ	*psbA, psbB, psbC, psbD, psbE, psbF, psbH, psbI, psbJ, psbK, psbL, psbM, psbN, psbT, psbZ*
	cytochrome b6f	*petA, petB, petD, petG, petL, petN*
	ATP synthase	*atpA, atpB, atpE, atpF, atpH, atpI*
	RuBisCo	*rbcL*
	NAD(P)H dehydrogenase genes	ψ*ndhA*, ψ*ndhB*, ψ*ndhC*, ψ*ndhD*, ψ*ndhE*, ~~*ndhF*~~, ψ*ndhG*, ψ*ndhH*, ~~*ndhI*~~, ψ*ndhJ*, ψ*ndhK*
RNA genes and genes for the genetic apparatus	transfer RNA	*trnH-GUG, trnK-UUU, trnQ-UUG, trnS-GCU, trnG-UCC, trnR-UCU, trnC-GCA, trnD-GUC, trnY-GUA, trnE-UUC, trnT-GGU, trnS-UGA, trnG-GCC, trnfM-CAU, trnS-GGA, trnT-UGU, trnL-UAA, trnF-GAA,* ~~*trnV-UAC*~~*, trnM-CAU, trnW-CCA, trnP-UGG*
	ribosomal RNA	*rrn4.5, rrn5, rrn16, rrn23*
	RNA polymerase	*rpoA, rpoB, rpoC1, rpoC2*
	Large ribosomal subunit genes	*rpl2, rpl14, rpl16, rpl20, rpl22, rpl23, rpl32, rpl33, rpl36*
	Small ribosomal subunit genes	*rps2, rps3, rps4, rps7, rps8, rps11, rps12, rps14, rps15, rps16, rps18, rps19,*
Potential protein-coding genes	Translational initiation factor	*infA*
	Maturase	*matK*
	Protease	*clpP*
	Envelop membrane protein	*cemA*
	Subunit of Acetyl-CoA-carboxylase	~~*accD*~~
	c-type cytochrom synthesis gene	*ccsA*
Conserved ORFs (ycfs)		*ycf1, ycf2, ycf3, ycf4,*

ψ indicate pseudogene, Strikethrough gene indicate gene loss.

Table S2. Blast result of plastid-derived DNA segments in the *Corydalis pauciovulata* mitogenome.

Mt-length	Pt-length	Identigy	Aligned length	Query start	Query end	Hit start	Hit end	E value	Bit-score	Annotated plastid genes
6500	6485	94.502	6530	29267	35751	232844	226345	0	10163	rpoB*, **rpoC1**, rpoC2*
2620	2646	95.342	2662	107667	110312	392857	395476	0	4241	16S rRNA*, **trnI**, trnA*
2419	2363	93.054	2433	103266	105628	43488	41070	0	3635	23S rRNA*, **4.5S rRNA**, **5S rRNA**, **trnR-ACG**
1668	1624	83.118	1700	69152	70775	92878	91211	0	1780	**psbE**, ψpsbF, **psbL**, **psbJ**, psaB*
1585	1610	79.514	1645	25804	27413	497649	499233	0	1443	atpI*, ψrps2, rpoC2*
1211	1185	75.922	1275	70893	72077	579649	580859	0	956	**petL**, **petG**, **trnW-CCA**, ψtrnP-UGC
1221	1232	95.536	1232	44287	45518	2293	1073	0	1975	psbD*, psbC*
1194	1194	95.645	1194	48216	49409	92892	94085	0	1920	psaB*
1120	1130	83.319	1151	125649	126778	410490	409371	0	1190	**ndhE**, ndhG*
1083	1078	90.511	1096	45781	46858	1084	2	0	1505	psbC*, **trnS-UGA**, **psbZ**
908	1031	76.731	1040	129840	130870	323447	324354	0	840	ycf1*
878	912	63.938	965	38979	39890	652571	651694	2.78E-36	154	**petN**
890	919	70.947	950	29449	30367	148993	148104	3.84E-123	444	rpoC2*
872	863	85.36	888	3222	4084	97181	98052	0	1017	matK*, trnK-UUU intron*
332	371	66.142	381	42757	43127	649827	649496	5.36E-20	101	trnT-GGU/psbD IGS*
315	324	88.58	324	48143	48466	48922	49236	1.53E-115	417	psaB*
288	312	78.275	313	44029	44340	431467	431180	9.06E-68	260	psbD*
301	290	74.434	309	39920	40209	650954	650654	2.00E-44	181	**psbM**
291	278	81.849	292	28440	28717	667193	667483	2.28E-75	284	rpoC2*
231	240	76.763	241	125046	125285	56112	55882	2.96E-42	175	ndhD*
222	229	80.426	235	87992	88220	384110	384331	2.28E-56	221	rpl14*
201	215	77.626	219	62013	62227	191676	191476	1.03E-41	172	ndhC*
194	195	92.821	195	48864	49058	191870	191677	6.52E-76	286	psaB*
119	151	72.185	151	35885	36035	579295	579177	1.87E-19	99.6	rpoB*
121	121	90.083	121	41186	41306	650156	650036	1.54E-39	165	**trnD-GUC**
94	95	77.778	99	4153	4247	213271	213178	2.14E-12	75.2	trnK-UUU intron*
83	83	92.771	83	115276	115358	351098	351180	4.70E-27	123	**trnN-GUU**
82	81	96.341	82	37	117	636210	636291	3.17E-29	132	**trnH-GUG**
80	80	88.75	80	47268	47347	26135	26056	4.40E-21	104	**trnfM-CAU**
68	75	81.333	75	94280	94354	653639	653706	2.14E-12	75.2	ψtrnI-CAU
73	73	94.521	73	13303	13375	163267	163195	2.44E-24	114	**trnM-CAU**
64	68	88.235	68	1622	1689	407658	407721	1.80E-17	86.9	ndhA*
439	453	88.742	453	1694	2146	407784	408222	1.78E-169	591	ndhA*
531	528	90.467	535	1	528	408301	408831	0	732	ψndhI
527	531	90.112	536	1	531	409204	409730	0	723	ψndhG
303	303	91.531	307	1	303	409977	410279	1.16E-123	435	**ndhE**

Bold fonts indicate intact genes, * indicates fragments, ψ indicate pseudogene

Table S3. Predicted RNA editing in 41 protein-coding genes for the *Corydalis pauciovulata* mitogenome.

	C. pauciovulata		*N. nucifera*		*L. tulipifera*	
Gene	Length (bp)	RNA editing	Length (bp)	RNA editing	Length (bp)	RNA editing
atp1	1,530	12	1,530	12	1,530	13
atp4	597	14	597	14	582	15
atp6	798	26	774	25	807	28
atp8	480	5	480	5	480	5
atp9	282	9	225	12	226	11
ccmB	621	40	621	41	621	41
ccmC	720	36	759	34	723	39
ccmFc	1,356	21	1356	21	1359	21
ccmFn	1,734	39	1734	41	1806	42
cob	1,212	22	1182	21	1182	28
cox1	1,584	34	1584	10	1584	36
cox2	786	17	783	18	759	17
cox3	798	19	798	20	798	21
matR	1,968	19	1968	18	1953	19
mttB	828	36	828	37	768	35
nad1	978	34	978	33	978	32
nad2	1,467	37	1467	37	1467	40
nad3	357	18	357	16	357	20
nad4	1,461	57	1488	14	1488	15
nad4L	303	15	303	63	303	61
nad5	2,001	39	3013	40	2013	44
nad6	738	20	633	17	723	24
nad7	1,188	40	1185	39	1185	39
nad9	573	14	573	15	573	14
rpl2	1,536	8	999	3	1665	6
rpl5	567	10	561	9	561	10
rpl10	489	6	489	7	480	8
rpl16	435	7	516	9	435	7
rps1	609	4	606	5	606	5
rps2	681	8	663	7	621	7
rps3	1,686	14	1692	13	1584	15
rps4	1,065	21	1059	21	1071	21
rps7	447	2	447	2	450	2
rps10	387	3	363	4	360	4
rps11	579	3	444	3	516	3
rps12	378	10	378	10	378	11
rps13	351	6	351	6	351	6
rps14	303	3	303	3	303	3
rps19	285	3	285	3	282	3
sdh3	336	2	375	2	330	8
sdh4	687	5	450	5	405	5
		738		715		784

Table S4. RNA editing validation of 21 genes using transcriptome data.

Gene	PREP-MT	> 0.5	TC	Gene	PREP-MT	> 0.5	TC	Gene	PREP-MT	> 0.5	TC	Gene	PREP-MT	> 0.5	TC
atp1	57		O	ccmC	458	0.78	O	cox2	676	1.00	O	nad9	92	0.75	O
	971	0.80	O		463	1.00	O		715	0.79	O		113	0.92	O
	1039	1.00	O		467	0.78	O		736	1.00	O		167	0.92	O
	1064	1.00	O		473	1.00	O		769	0.50			190	1.00	O
	1115	1.00	O		497	1.00	O	cox3	112	1.00	O		223	1.00	O
	1168	1.00	O		499	1.00	O		245	0.92	O		298	0.83	O
	1178	0.90	O		521	1.00	O		257	0.67	O		311	0.75	O
	1216	1.00	O		548	1.00	O		263	1.00	O		328	1.00	O
	1262	1.00	O		568	1.00	O		289	0.92	O		368	1.00	O
	1292	0.80	O		572	1.00	O		298	1.00	O		398	1.00	O
	1415	1.00	O		575	1.00	O		304	1.00	O		406	1.00	O
	1490	0.90	O		605	1.00	O		311	0.92	O		439	1.00	O
	1499	0.90	O		608	0.89	O		314	0.92	O		539	1.00	O
atp4	56	0.86	O		614	0.78	O		388	1.00	O	rpl10	77	1.00	
	59	0.57	O		619	0.78	O		413	0.83	O		83		O
	71	1.00	O		650	0.78			419	1.00	O		101	0.83	O
	89	1.00	O		656	0.89			422	0.92	O		134	0.83	O
	118	0.71	O		665	0.78			512	0.75	O		155	0.83	O
	121	1.00	O		673	0.78			527	0.92	O		314	0.83	O
	215	1.00	O	cob	118	0.92	O		566	0.92	O		397		O
	226	0.57			178	1.00	O		653	1.00	O		409	0.83	
	227	0.57	O		286	1.00	O		754	0.92	O	rps1	26		O
	248	1.00	O		298	1.00	O		764	0.92			45		O
	250		O		325	1.00	O	matR	26	1.00	O		74	1.00	O
	251		O		358	1.00	O		32	0.62	O		128	0.67	O
	395	1.00	O		407	1.00	O		43		O		161	0.67	O
	407	0.71	O		419	1.00	O		193	0.75	O		212		O
	416	0.86	O		564		O		235	0.62	O		485	0.67	
	490	0.57			568	0.92	O		236	0.62	O		590		O
atp6	37	0.75	O		580	1.00	O		239	0.75		rps3	58	1.00	O
	47	1.00	O		680	1.00	O		249		O		64	1.00	O
	116	1.00	O		715	1.00	O		326	1.00	O		69		O
	167	1.00	O		725	1.00	O		382	0.75	O		92	0.71	O
	173	1.00	O		808	1.00	O		413		O		126		O
	224	1.00	O		853	1.00	O		923	1.00			512	0.71	O
	229	0.75	O		908	1.00	O		1009	0.88			599	1.00	O
	236	0.67	O		914	1.00	O		1064	1.00	O		699		O
	254	1.00	O		982	0.85			1065		O		713	0.86	O
	262	1.00	O		1015	1.00	O		1675	1.00	O		1031	0.86	O
	269	1.00	O		1081	0.77	O		1679	1.00	O		1364	1.00	O
	270		O		1084	1.00	O		1700	1.00	O		1391	0.86	O
	337	1.00	O		1124	1.00	O		1720	1.00	O		1502	1.00	O
	401	1.00	O	cox1	**2**		**O**		1734		O		1555	1.00	O
	460	1.00	O		11		O		1756	1.00	O		1588	0.57	
	463	1.00	O		15		O		1787	1.00	O		1589		O
	485	1.00	O		196	1.00	O		1826	0.88	O		1619	0.86	O
	527	1.00	O		197	1.00	O		1844	0.88	O		1633	1.00	O
	548	1.00	O		242	1.00		mttB	44		O	rps4	5		O
	599	0.92	O		254	1.00			55		O		50	1	O
	635	1.00	O		299	1.00	O		65	0.88	O		55		O
	656	1.00	O		352	1.00	O		76		O		56	0.5	O
	664	1.00	O		443	1.00	O		98		O		67	1	O
	671	1.00	O		452	1.00	O		103	0.88	O		151	0.67	O
	680	1.00	O		458	1.00	O		115		O		182	1	O
	707	0.92	O		515	1.00	O		139	0.88	O		202	0.83	O
	**718**	**1.00**	**O**		551	1.00	O		151	1.00	O		211	1	O
atp8	21		O		575	1.00	O		161	0.50	O		275	1	O
	30		O		590	1.00	O		167	0.88	O		284	0.83	O
	47	1.00	O		668	1.00	O		170	1.00	O		296	0.67	O
	55	0.75			715	1.00	O		217	1.00	O		308	0.83	O
	58	1.00	O		746	1.00	O		227	0.75	O		325	0.5	O
	60		O		761	1.00	O		239	0.62	O		353	1	O
	76		O		845	1.00	O		241	0.88	O		457		O
	200	1.00	O		860	1.00	O		263	0.50	O		482		O
	273		O		868	1.00	O		291		O		500	1	O
	432		O		896	1.00	O		301	1.00	O		932	0.83	
	452	0.75	O		1037	1.00	O		320	0.75	O		943	0.83	
atp9	20	1.00	O		1079	1.00	O		367	0.88	O		953	0.67	
	50	1.00	O		1124	1.00	O		370	0.62	O		968	1	
	82	1.00	O		1165	1.00	O		383	0.75	O		1018		O
	90		O		1186	1.00	O		385	0.88	O		1019	0.83	O
	92	1.00	O		1259	1.00	O		392	0.50			1028	1	O
	134	1.00	O		1274	0.89	O		412		O		1033	1	O
	182	1.00	O		1279	1.00	O		413		O	rps7	116	0.75	
	191	1.00	O		1373	0.89			415	0.50	O		332	0.88	O

	205		O		1402	0.67	O		418	0.62	O	rps12	71	0.94	O
	212	1.00	O		1405	0.78	O		445		O		100	1	O
	223	1.00	O		1433	1.00	O		446	0.75	O		104	1	O
ccmC	5		O		1446		O		453		O		112	0.88	O
	103	1.00	O		1489	1.00	O		476	0.57	O		143		O
	115	0.78	O	cox2	27		O		511	0.88	O		146	1	
	128	0.56			33		O		536	0.88	O		148		O
	133	0.67	O		38		O		544	1.00	O		196	0.94	O
	179	0.78	O		71	1.00	O		580	0.88	O		221	0.88	O
	184	1.00	O		161	0.95	O		587	0.62	O		232	1	O
	227	0.78	O		253	1.00	O		593	0.50	O		269	0.94	O
	281	0.56	O		278	1.00	O		617	0.75	O		284	0.76	O
	299	1.00	O		379	1.00	O		649	1.00	O	rps13	5	0.6	O
	331	1.00	O		443	1.00	O		699		O		26	0.9	O
	358	1.00	O		460		O		706	0.62	O		56	0.9	O
	395	1.00	O		461	1.00	O		711		O		100	0.9	O
	399		O		476	1.00	O		712		O		256	1	O
	400	0.89	O		544	1.00	O		713	0.50			287	1	
	421	0.78	O		557	1.00	O		722	0.50	O	sdh3	54		O
	436	0.89	O		581	1.00	O		743	1.00	O		67	1	O
	446	0.78	O		614	1.00	O		752	0.75	O		74	1	O
	451	1.00	O		632	0.84	O	nad9	14	0.73	O				

Table S5. Blast results of ORFs (>150 bp) in the *Corydalis pauciovulata* mitogenome.

Name	aa	Length	Min	Max	E-Value	Bitscore	Accession	Short name
ORF115	115	348	17,249	16,902	0.00030787	37.241	cl21549	rve superfamily
ORF283	283	852	34,730	35,581	0.00373583	37.6768	cl24149	MCPVI superfamily
ORF94	94	285	44,127	44,411	4.44E-06	42.2746	cl37957	RVT_1 superfamily
ORF126	126	381	61,235	60,855	3.36E-18	76.4708	cl02808	RT_like superfamily
ORF71	71	216	63,098	62,883	0.00514233	33.0558	cl04955	LanC_like superfamily
ORF68	68	207	124,460	124,666	6.96E-03	32.6041	cl00467	Ntn_hydrolase superfamily
ORF192	192	579	147,778	147,200	5.06E-34	116.823	cd09274	RNase_HI_RT_Ty3
ORF53a	53	162	166,866	166,705	1.95E-03	32.6155	cl16506	zf-RVT superfamily
ORF102	102	309	192,760	192,452	1.10E-08	47.2757	pfam17921	Integrase_H2C2
ORF97a	97	294	219,704	219,997	1.71E-04	38.3459	cl28114	MatK_N superfamily
ORF62	62	189	221,790	221,602	0.00681989	31.6238	cl00075	HATPase superfamily
ORF67	67	204	224,063	224,266	1.17E-20	81.0079	cl06662	RVT_2 superfamily
ORF117a	117	354	224,227	224,580	4.85E-25	94.4899	cl06662	RVT_2 superfamily
ORF235a	235	708	225,328	226,035	2.49E-06	47.3813	cl05469	Mitovir_RNA_pol superfamily
ORF252	252	759	254,757	255,515	7.77E-13	65.718	pfam03372	Exo_endo_phos
ORF235b	235	708	255,825	256,532	6.50E-16	73.4794	cl02808	RT_like superfamily
ORF201	201	606	261,261	261,866	1.41E-04	41.6033	cl05469	Mitovir_RNA_pol superfamily
ORF154	154	465	264,904	265,368	1.62E-21	84.185	cl16623	DUF4283 superfamily
ORF208	208	627	265,788	266,414	0.00647206	36.1753	cl00490	EEP superfamily
ORF121	121	366	272,489	272,124	1.86E-03	33.304	cl24970	zf-C2H2_10 superfamily
ORF100a	100	303	281,917	281,615	3.61E-11	56.9158	cl02808	RT_like superfamily
ORF111	111	336	293,729	293,394	1.44E-21	84.56	cl02808	RT_like superfamily
ORF117b	117	354	294,271	293,918	3.73E-17	75.5008	cl05469	Mitovir_RNA_pol superfamily
ORF178	178	537	322,474	321,938	9.75E-27	104.733	cl28058	DNA_pol_B_2 superfamily
ORF110a	110	333	335,020	334,688	1.90E-06	43.0479	cl07848	ELF superfamily
ORF117c	117	354	336,406	336,053	5.23E-03	33.0234	pfam17921	Integrase_H2C2
ORF87	87	264	356,456	356,719	7.39E-31	106.526	cd01647	RT_LTR
ORF63	63	192	363,019	362,828	4.63E-03	33.0334	cl02808	RT_like superfamily
ORF234	234	705	368,537	369,241	6.89E-05	40.3515	cl00350	Ribosomal_S19 superfamily
ORF128	128	387	379,319	379,705	8.16E-21	83.4044	cl02808	RT_like superfamily
ORF54	54	165	451,349	451,513	0.0030072	31.1294	cl18687	zf-CCHC_4 superfamily
ORF123	123	372	461,763	461,392	3.72E-41	132.137	cl19078	REC superfamily
ORF75	75	228	461,962	461,735	8.99E-08	45.527	cl00075	HATPase superfamily
ORF57	57	174	462,552	462,379	7.41E-07	41.0154	pfam00512	HisKA
ORF79	79	240	463,305	463,066	2.44E-13	60.1745	cl14782	RNase_H_like superfamily
ORF100b	100	303	473,907	473,605	0.00668953	34.2415	cl17011	Arginase_HDAC superfamily
ORF55	55	168	476,720	476,553	8.19E-03	31.877	cl21496	2OG-FeII_Oxy superfamily
ORF155	155	468	478,020	478,487	2.90E-18	77.6264	cl02808	RT_like superfamily
ORF93	93	282	492,067	492,348	3.78E-19	75.2217	cl14782	RNase_H_like superfamily
ORF97b	97	294	500,786	500,493	2.32E-03	35.2798	cl19167	Bac_export_2 superfamily
ORF106	106	321	506,331	506,011	0.00640333	34.1905	cl06214	FYTT superfamily
ORF119	119	360	509,910	510,269	6.91E-28	99.2078	pfam14111	DUF4283
ORF333	333	1,002	528,991	529,992	6.08E-71	218.234	cd01647	RT_LTR
ORF127	127	384	530,032	530,415	1.89E-14	63.6471	pfam17919	RT_RNaseH_2
ORF167	167	504	530,351	530,854	4.25E-14	64.8213	cl14782	RNase_H_like superfamily
					0.00298028	35.9113	cl14782	RNase_H_like superfamily
ORF226	226	681	530,863	531,543	4.37E-17	74.4312	cl14782	RNase_H_like superfamily
					6.38E-04	36.8753	pfam17921	Integrase_H2C2
ORF148	148	447	531,583	532,029	1.12E-14	65.3605	pfam00665	rve
ORF539	539	1,620	534,606	532,987	4.12E-04	41.899	cl37731	BASP1 superfamily
ORF145	145	438	534,992	534,555	4.81E-31	113.564	cl39183	PMD superfamily
ORF110b	110	333	545,805	546,137	5.59E-08	46.4036	cl21610	PQ-loop superfamily
ORF125	125	378	575,016	574,639	1.08E-07	45.0354	cl39048	Retrotran_gag_3 superfamily
ORF107	107	324	576,313	575,990	1.24E-44	141.066	cd09272	RNase_HI_RT_Ty1
ORF88	88	267	576,612	576,346	1.58E-04	37.4477	cl14782	RNase_H_like superfamily
ORF244	244	735	592,626	591,892	1.80E-12	61.5792	pfam03732	Retrotrans_gag
ORF53b	53	162	663,285	663,124	7.44E-03	32.0115	cl34545	PilF superfamily

Table S6. Potential chimeric ORFs.

	ORF start	ORF end	ORF length	Identity	ORF hit start	ORF hit end	Chimera length	Gene hit start	Gene hit end	E-value	Gene	No of Transmembrane helices / probabilities	Transcript
**ORF457a**	**593255**	**594628**	**1374**	**99.7**	**1**	**701**	**701**	**1**	**701**	**0.00E+00**	***atp1***	**1 / 0.72285**	○
**ORF457b**	655980	654607											
**ORF244**	**259778**	**260512**	**735**	**89.5**	**19**	**56**	**38**	**1**	**38**	**6.38E-09**	***rpl2***	**1 / 0.99742**	○
**ORF234**	**368537**	**369241**	**705**	**84.1**	**1**	**79**	**82**	**1**	**82**	**8.50E-20**	***rps19***	**1 / 0.97547**	○
**ORF146**	**258635**	**259075**	**441**	**94.3**	**94**	**128**	**35**	**307**	**341**	**3.10E-10**	***rpl5***	**3 / 0.13557**	○
ORF112	528026	527688	339	96.8	138	168	31	303	283	2.87E-09	*ccmFc*	0 / 0.48217	
ORF60a	246603	246785	183	100	31	93	63	447	385	1.49E-28	*rps7*	0 / 0.34003	
ORF60b	633132	632950	183	82.1	140	177	39	718	756	1.15E-04	*cob*	0 / 0.21732	
				96.8	136	166	31	274	304	1.49E-09	*nad5*		
**ORF56**	**239013**	**239183**	**171**	**95.4**	**84**	**148**	**65**	**681**	**745**	**2.50E-25**	***cob***	**1 / 0.15049**	
**ORF54**	**154633**	**154469**	**165**	**97.1**	**1**	**34**	**34**	**1**	**34**	**3.13E-11**	***sdh3***	**1 / 0.04103**	○
ORF49a	45477	45328	150	90.3	27	57	31	241	271	6.20E-07	*cob*	0 / 0.42089	
ORF49b	353025	351876	150	100	74	103	30	610	639	9.82E-11	*sdh4*	0 / 0.51849	○

Table S7. Information on the nuclear-encoded NDH complex genes.

Group	Gene	Accession number		
		Arabidopsis thaliana	*Aquilegia coerulea*	*Corydalis ochotensis*
Linkers	LHCA5	AT1G45474	Aqcoe6G141900.1	○
	LHCA6	AT1G19150	Aqcoe7G431400.1	○
subcomplex A	NdhL	AT1G70760	Aqcoe3G005000.1	-
	NdhM	AT4G37925	Aqcoe6G031500.1	-
	NdhN	AT5G58260	Aqcoe5G227700.1	-
	NdhO	AT1G74880	Aqcoe5G236000.1	-
subcomplex EDB	NdhS	AT4G23890	Aqcoe2G163300.1	-
	NdhT	AT4G09350	Aqcoe6G015200.1	○
	NdhU	AT5G21430	Aqcoe7G114900.1	-
	NdhV	AT2G04039	Aqcoe1G375600.1	-
Subcomplex B	PnsB1	AT1G15980	Aqcoe7G199900.1	-
	PnsB2	AT1G64770	Aqcoe2G064400.1	-
	PnsB3	AT3G16250	Aqcoe7G162000.1	○
	PnsB4	AT1G18730	Aqcoe3G060000.1	-
	PnsB5	AT5G43750	Aqcoe5G042700.1	-
subcomplex L	PnsL1	AT2G39470	Aqcoe1G473000.1	○
	PnsL2	AT1G14150	Aqcoe3G060200.1	○
	PnsL3	AT3G01440	Aqcoe1G367800.1	○
	PnsL4	AT4G39710	Aqcoe6G058500.1	○
	PnsL5	AT5G13120	Aqcoe3G164000.1	○

○: present, -: absent

Table S8. Information on the nuclear-encoded DNA-RRR genes.

Category	gene	TAIR ID	Targeting	*Liriodendron tulipifera*	*Nelumbo nuicifera*	*Corydalis pauciovulata*			
						GenBank accession numbers	CDS	length	exon
DNA polymerase	POLIA	AT1G50840	plastid/mitochondria	O	O	PP437511	3339	8,383	13
	POLIB	AT3G20540	plastid/mitochondria	-	-	-	-	-	-
Helicase/primase	Twinkle	AT1G30680	plastid/mitochondria	O	O	PP437512	2130	28,267	21
Topoisomerase	GYRA	AT3G10690	plastid/mitochondria	O	O	PP437513	2910	26,767	27
	GYRBC	AT3G10270	plastid	O	O	PP437514	2253	2,253	1
	GYRBM	AT5G04130	mitochondria	-	-	-	-	-	-
	TopoI	AT4G31210	plastid/mitochondria	O	partial/XM_010259587	PP437515	3618	14,933	22
Recombinase	RECA1	AT1G79050	plastid	O	O	PP437516	1257	18,399	13
	RECA2	AT2G19490	plastid	O	partial/XM_010252152	PP437517	1278	3,097	8
	RECA3	AT3G10140	mitochondria	O	O	PP437518	1218	4,875	8
	RECG	AT2G01440	plastid/mitochondria	O	partial/XM_010276366	PP437519	3189	33,633	17
	RECX	AT3G13226	plastid/mitochondria	O	O	PP437520	999	2,494	6
ssDNA-binding/recombination mediator	SSB1	AT4G11060	mitochondria	O	O	PP437521	684	8,003	4
	SSB2	AT3G11060	mitochondria	-	-	-	-	-	-
	OSB1	AT1G47720	mitochondria	O	O	PP437522	900	1,846	4
	OSB2	AT4G20010	plastid	O	O	PP437523	1245	13,149	8
	OSB3	AT5G44785	plastid/mitochondria	-	-	-	-	-	-
	OSB4	AT1G31010	mitochondria	-	-	-	-	-	-
	WHY1	AT1G14410	plastid	O	O	PP437524	804	3,261	7
	WHY2	AT1G71260	mitochondria	O	O	PP437525	738	4,787	8
	WHY3	AT2G02740	plastid	-	O	-	-	-	-
	ODB1	AT1G71310	mitochondria	O	O	PP437526	612	3,383	4
	ODB2	AT5G47870	plastid	O	O	PP437527	687	10,426	4
MutS	MSH1	AT3G24320	plastid/mitochondria	partial	partial/XM_019198676	PP437528	3378	46,688	22
DNA glycosylase	UNG	AT3G18630	mitochondria	partial	O	PP437529	966	4,031	7
	OGG1	AT1G21710	nucleus/plastid	O	O	PP437530	1206	2,824	3
	NTH1	AT2G31450	plastid	O	O	PP437531	1053	3,963	11
	NTH2	AT1G05900	Plastid/Mitochondria	-	-	-	-	-	-
AP endonuclease	ARP	AT2G41460	plastid	O	partial/XM_010275015	PP437532	1668	5,471	13
	APE1L	AT3G48425	nuclear/plastid	O	O	PP437533	854	5,187	9
	APE2	AT4G36050	nuclear/mitochondria	O	partial/XM_010270362	PP437534	1947	3,671	10
DNA ligase	LIG1	AT1G08130	nuclear/mitochondria	O	O	PP437535	2451	5,591	17

○: present, -: absent
